# Palbociclib targets β-catenin for degradation and synergizes with KRAS or ERK5 inhibition in colorectal cancer preclinical models

**DOI:** 10.1186/s12967-026-08420-7

**Published:** 2026-06-19

**Authors:** Matteo Villa, Federica Malighetti, Alberto Maria Villa, Nicoletta Cordani, Andrea Aroldi, Alfonso Zambon, Daniele Ramazzotti, Luca Mologni

**Affiliations:** 1 Department of Medicine and Surgery, University of Milano-Bicocca, 20900 Monza, Italy; 2 Fondazione IRCCS San Gerardo dei Tintori, 20900 Monza, Italy; 3 Department of Chemistry and Geological Sciences, University of Modena and Reggio Emilia, 41125 Modena, Italy

**Keywords:** Colorectal cancer, Palbociclib, WNT/β-catenin signaling, KRAS, ERK5

## Abstract

**Background:**

Colorectal cancer (CRC) is a leading cause of cancer-related mortality, largely driven by aberrant activation of the WNT/β-catenin and RAS pathways. Despite recent therapeutic advances, effective strategies to target these oncogenic signals remain limited.

**Methods:**

A multi-step chemical library screen was performed to identify compounds capable of suppressing β-catenin and RAS signaling. Drug mechanisms and combination effects were investigated through functional assays, in vitro and in vivo experiments, and transcriptomic profiling. Survival analysis of the Sidra-LUMC AC-ICAM CRC cohort (*n* = 303) was conducted to evaluate the prognostic significance of candidate target genes.

**Results:**

We identified the CDK4/6 inhibitor palbociclib as an unexpected suppressor of β-catenin signaling. Uniquely, palbociclib promoted GSK3β-mediated β-catenin degradation by dampening AKT activity, revealing a previously unrecognized mechanism of action and broadening its role beyond canonical cell-cycle inhibition. Building on this mechanism, we found that combining palbociclib with the KRAS^G12D^-specific inhibitor MRTX1133 elicited potent and selective anti-tumor effects in vitro and in vivo. Parallel screening also revealed the ERK5 inhibitor ERK5-IN-1 as a promising combination partner: co-administration with palbociclib strongly suppressed proliferation across multiple CRC models, showed minimal toxicity in normal cells, and produced durable tumor control in vivo. Transcriptomic profiling indicated that both combinations converge on a common program of cancer cell-state reprogramming, characterized by suppression of proliferative drivers and remodeling of metabolic and mitochondrial pathways. Underscoring the clinical relevance of our findings, survival analysis of the Sidra-LUMC AC-ICAM CRC cohort (*n* = 303) revealed that ERK5 target genes *CREB5* and *NUPR1*, identified in our dataset, were consistently linked to poor prognosis, thereby connecting this signaling axis to unfavorable patient outcomes.

**Conclusions:**

Together, these findings position palbociclib as a versatile therapeutic backbone in CRC. By simultaneously targeting cell cycle and oncogenic signaling networks, palbociclib-based combinations induce synergistic and durable responses, offering a compelling rationale for tailored therapeutic strategies in molecularly defined CRC.

**Supplementary Information:**

The online version contains supplementary material available at https://doi.org/10.1186/s12967-026-08420-7.

## Background

Colorectal cancer (CRC) is one of the most prevalent and lethal malignancies worldwide, ranking as the third most common cancer and the second leading cause of cancer-related deaths globally [1]. While the incidence and mortality have decreased in countries where screening is widespread, there has been an increase in young adults [2]. The development of CRC is a complex, multistep process involving genetic mutations, epigenetic changes, and environmental factors. Cancer stem cells play a crucial role in tumor initiation and therapeutic resistance [3]. Most colorectal cancers begin as benign adenomatous polyps that can progress to malignancy over several years [4] due to the accumulation of several genetic alterations. CRC can arise through several genetic mechanisms, including chromosomal instability, microsatellite instability and CpG island methylator phenotype [5]. These CRC subtypes often show dysregulation of several signaling pathways such as NF-κB, Wnt/β-catenin, Ras/MAPK, p53 and PI3K/AKT [6, 7]. In particular, key mutations frequently observed in CRC include those in the APC, KRAS and TP53 genes, which contribute to the dysregulation of cellular pathways involved in proliferation, apoptosis and genomic stability [8].

Among these, the Wnt/β-catenin and Ras signaling pathways have been implicated both in tumor progression and in resistance to therapy. The Wnt/β-catenin pathway is altered in nearly 90% of CRC cases [9]. Its aberrant activation results in the accumulation and nuclear translocation of β-catenin, which in turn drives the transcription of genes that promote tumor growth and metastasis [10, 11]. Elevated β-catenin activity is associated with increased malignancy and poor prognosis in CRC patients, making it a prime therapeutic target and a promising avenue for treatment strategies [12]. Similarly, KRAS mutations are present in approximately 40% of CRC cases and are associated with aggressive tumor behaviour and resistance to standard therapies, including anti-EGFR agents. KRAS mutations lead to constitutive activation of the RAS/RAF/MEK/ERK pathway, which further stimulates tumor cell proliferation, survival, and metastasis [13, 14]. Given the challenges associated with directly targeting mutant KRAS, recent therapeutic strategies have focused on inhibiting downstream effectors and parallel pathways to reduce tumor progression [15, 16]. Recent advancements in KRAS-specific inhibitors and combination therapies show promise in addressing adaptive resistance mechanisms [14]. Notably, around 30–60% of CRC samples carry mutations in both pathways simultaneously [17, 18] implying that concurrent blockade of both mechanisms is needed to achieve effective tumor growth inhibition.

Current therapeutic options for CRC include surgery, chemotherapy, radiotherapy, and targeted therapies against VEGF and EGFR [4, 5]. Despite advancements, the prognosis for CRC patients, especially those with metastatic disease, remains poor [4]. Thus, a deeper understanding of the molecular mechanisms driving CRC progression and resistance to therapy is essential for the development of new and more effective treatment strategies. Emerging research focuses on alternative therapies and the development of biomarker panels for improved prognosis and treatment selection [5, 7].

In our previous work, we showed that combined shRNA-mediated silencing of β-catenin and KRAS in CRC cells resulted in a strong induction of apoptosis in vitro and significant tumor growth suppression in vivo, whereas targeting either pathway individually produced only modest effects [19]. Building on these findings, a proof-of-concept study was then conducted employing a pharmacological combination strategy. Unfortunately, the effects of these combinations could not be tested in mice due to technical limitations of the drugs [20]. Indeed, to date, no clinical grade β-catenin inhibitor has been identified. Moreover, given the high genetic heterogeneity of CRC and the involvement of multiple oncogenic drivers beyond β-catenin and KRAS, a broader approach is warranted. In this study, we employed a drug library screening strategy to identify compounds that could show therapeutic potential in this context. We found that the CDK4/6 inhibitor palbociclib induces β-catenin protein downregulation in CRC cells and synergizes with KRAS inhibition. In addition, we show potent synergistic tumor growth inhibition using palbociclib combined with an ERK5 inhibitor. Our results have potential implications for improved outcomes in CRC patients.

## Methods

### Cell lines and drugs

The SW837 cell line was a kind gift from Dr. Manuela Gariboldi (Fondazione IRCCS Istituto Nazionale dei Tumori di Milano, Milano, Italy), who originally obtained it from the American Type Culture Collection (ATCC). Cell identity was confirmed by microsatellite genotyping at the IFOM Cell Biology Unit. All other cell lines were purchased from ATCC, where routine genotypic and phenotypic testing ensures their authentication. Ls174T and HCT116 cells harbor mutations in the CTNNB1 gene that lead to stabilization of β-catenin [21], whereas DLD-1, SW480, and SW837 cells carry truncating mutations in the APC gene [22, 23]. All five cell lines also express constitutively active KRAS protein due to oncogenic mutations [23–25]. A detailed list of mutations is provided in Supplementary Table 1. Ls174T cells stably transfected with doxycycline-inducible shRNA constructs targeting CTNNB1 or KRAS were previously described [19]. HEK-293 and RWPE1 cell lines were used as non-tumorigenic controls to assess treatment specificity and toxicity. All cell lines were maintained in RPMI 1640 medium, except for DLD-1 and HEK-293 cells, which were cultured in DMEM. Media were supplemented with 10% fetal bovine serum (FBS), 100 U/mL penicillin, 100 µg/mL streptomycin, and 2 mM L-glutamine. Cells were incubated at 37 °C in a humidified atmosphere containing 5% CO₂. The Kinase Inhibitor Library (cat# 1200) was purchased from Selleck Chemicals. All individual compounds were purchased from MedChemExpress and dissolved in DMSO or water according to the manufacturer’s datasheet at a stock concentration of 10 mM. Aliquots were stored at − 80 °C until use.

### Murine xenograft model and treatments

Twenty-eight six-week-old female athymic Nude mice (nu/nu) were purchased from Envigo (Milan, Italy) and maintained under standard housing conditions, in accordance with institutional guidelines and protocols approved by the University of Milano-Bicocca Ethical Committee for Animal Welfare and the Italian Ministry of Health. Pilot tests indicated that ERK5-IN-1 should be dosed below 50 mg/kg on an intermittent schedule to avoid systemic toxicity. For efficacy experiments, a total of 5 × 10⁶ Ls174T cells suspended in 0.2 mL PBS were subcutaneously injected into the right flank of each mouse. Once tumors reached an average volume of approximately 100 mm³, animals were randomly assigned to one of the following treatment groups: Vehicle control (10% DMSO in saline), palbociclib, 75 mg/kg dissolved in 10% DMSO/90% saline, administered daily via oral gavage; ERK5-IN-1 40 mg/kg dissolved in 30% β-cyclodextrin (w/v) in saline, administered intraperitoneally for 2 consecutive days followed by 2 days off, in repeated cycles; MRTX1133 10 mg/kg in 50 mM sodium citrate containing 10% β-cyclodextrin (w/v), injected intraperitoneally twice daily; palbociclib+ERK5-IN-1; palbociclib+MRTX1133. Treatment with palbociclib and ERK5-IN-1 was continued for 15 days, while MRTX1133 was administered for 10 days. Mice were monitored daily for signs of toxicity or distress. Tumor size was measured daily using a caliper, and tumor volumes were calculated using the formula: Tumor volume (mm³) = 𝑑^2^×𝐷/2, where *d* is the shortest and *D* is the longest tumor diameter (in mm). Event-free survival (EFS) was estimated by Kaplan-Meier method and EFS curves were compared by Log-rank test. An event was defined as the time to tumor doubling (200 mm³).

### Cell proliferation

Ten thousand cells per well were seeded in triplicate in 96-well plates. For dose–response experiments, cells were treated with vehicle (0.1% DMSO) or increasing concentrations of the indicated drugs for 72 h. Cell viability was assessed using the CellTiter 96® AQueous One Solution Cell Proliferation Assay (Promega, Madison, WI, USA) according to the manufacturer’s instructions. Absorbance at 490 nm was measured using an Infinite F200 PRO microplate reader (Tecan). Proliferation curves and IC₅₀ values were generated using GraphPad Prism software (GraphPad Software, Inc.). For time-course experiments, the cells were seeded at low density and harvested at each time point for MTS assay. Treatment was maintained by replacing the culture medium and administering fresh drug every two days. In addition, daily images were acquired to assess confluence and correlate morphological growth with MTS-based viability data.

### Synergism analysis

Cells were treated with vehicle or with inhibitors, either as single agents or in combination, at different concentrations and ratios. After 3 days of treatment, cell growth and viability were assessed using the MTS assay. Relative growth compared to vehicle-treated controls was used to calculate the fraction affected for each condition. Drug interaction effects were evaluated using the Bliss independence model, and synergy scores were calculated accordingly as the percent deviation from the expected additivity (excess over Bliss), using the formula: (S_ABobs_-S_ABexp_)/S_ABexp_, where S_AB_ is the surviving fraction with the combination, observed (obs) and expected (exp). S_ABexp_ is the product of the individual effects of each drug alone (S_A_×S_B_). When the observed survival of a combination is lower than expected assuming independent action, the combination is considered synergistic. Bliss scores < -0.10 were considered synergistic.

### Luciferase assays

Transcriptional activity of β-catenin in Ls174T cells was assessed using a dual-luciferase reporter system. Cells were seeded in 6-well plates and treated for 72 h with the indicated inhibitors or vehicle control. After treatment, cells were co-transfected with 1 µg of the TopFlash plasmid, containing six TCF4-binding sites upstream of a firefly luciferase reporter, and 0.1 µg of the phRL-CMV plasmid encoding renilla luciferase for normalization of transfection efficiency. Twenty-four hours post-transfection, cells were lysed and luciferase activity was measured using the Dual-Luciferase® Reporter Assay System (Promega), following the manufacturer’s protocol. Firefly and renilla luciferase signals were recorded using an Infinite F200 PRO microplate reader (Tecan), and firefly luciferase activity was normalized to renilla luciferase values.

### Quantitative real-time PCR

Cells were treated with vehicle or inhibitors for 72 h and then harvested. Total RNA was extracted using TRI Reagent (Sigma-Aldrich) according to the manufacturer’s instructions and reverse-transcribed into cDNA using the TaqMan™ Reverse Transcription Reagents (Invitrogen). Quantitative real-time PCR (qPCR) was performed using TaqMan probe-based assays (Thermo Fisher Scientific) for the analysis of β-catenin (Assay ID: Hs00355045_m1), Axin2 (Assay ID: Hs00610344_m1), c-Myc (Assay ID: Hs00153408_m1), CCND1 (Assay ID: Hs00765553_m1) in combination with the Luna® Universal Probe qPCR Master Mix (New England Biolabs). Reactions were run on an AriaMx Real-Time PCR System (Agilent) under the following cycling conditions: initial denaturation at 95 °C for 10 min, followed by 43 cycles of 95 °C for 15 s and 60 °C for 1 min. The β-glucuronidase (GUS) gene was used as an internal reference, and relative gene expression was calculated by normalizing target gene expression to GUS.

### RNA sequencing and data analysis

Ls174T cells were treated with 0.1% DMSO vehicle, 3 µM palbociclib, 10 µM MRTX1133, 1.25 µM ERK5-IN-1, or their combinations, palbociclib+MRTX1133 and palbociclib+ERK5-IN-1, for 72 h. Total RNA was extracted from cells using TRI Reagent (Sigma-Aldrich) according to the manufacturer’s instructions. For each condition, three independent cell populations were collected. RNA-seq libraries were prepared using the TruSeq Stranded mRNA Library Prep Kit (Illumina, Milan, Italy) and sequenced by Genewiz (Azenta Life Sciences, Leipzig, Germany) in paired-end mode (2 × 150 bp), with a minimum depth of 20 million read pairs per sample. Raw sequencing reads were aligned to the human reference genome (GRCh38/hg38) using HISAT2. Differential gene expression analysis was performed with the DESeq2 package [26]. Functional enrichment analysis of differentially expressed genes was carried out using WebGestalt [27]. Gene Set Enrichment Analysis (GSEA) was performed using normalized counts, with 1,000 permutations per comparison. An FDR < 0.05 was considered statistically significant. z-score normalization was applied according to the formula: z = (x − µ) / σ, where x is the raw value, µ is the mean, and σ is the standard deviation.

### Western blotting

Cells were lysed in Laemmli buffer supplemented with β-mercaptoethanol and heated at 95 °C for 20 min. Denatured protein samples were resolved by SDS–PAGE and transferred onto nitrocellulose membranes (Amersham™ Protran^®^ 0.45 μm NC, GE Healthcare Life Sciences). After blocking, membranes were incubated overnight at 4 °C with the appropriate primary antibodies. The following day, HRP-conjugated secondary antibodies were added for 1 h at room temperature. Protein signals were detected by chemiluminescence using Westar Nova 2.0 reagents (Cyanagen) and visualized with the ChemiDoc XRS+ imaging system (Bio-Rad). Band intensity was quantified using the Image Lab software (Bio-Rad). The primary antibodies used were: β-catenin (BD Biosciences, cat# 610154; dilution (dil) 1:1000), phospho-β-catenin (Ser33/Ser37/Thr41; Cell Signaling Technology [CST], cat# 9561; dil 1:1000), phospho-Akt (Ser473; CST, cat# 4060; dil 1:2000), phospho-Akt (Thr308; CST, cat# 13038; dil 1:1000), phospho-Akt (Ser129; CST, cat# 13461; dil 1:1000), Akt (CST, cat# 9272; dil 1:1000), phospho-GSK3β (Ser9; CST, cat# 9336; dil 1:1000), GSK3β (CST, cat# 9315; dil 1:1000), phospho-Rb (Ser807/811; CST, cat# 9308; dil 1:1000), phospho-Rb (Ser780; Cusabio, cat# CSB-PA133410; dil 1:500), Rb (Abcam, cat# ab24; dil. 1:500), phospho-RPS6 (Ser240/244; CST, cat# 2215; dil. 1:1000), phospho-RPS6 (Ser235/236; CST, cat# 4858; dil. 1:2000), RPS6 (Santa Cruz, cat# sc-74459; dil. 1:500), c-Myc (CST; cat# 9402; dil. 1:1000), CCND1 (Daco; cat# M3642; dil. 1:200), p21 (CST; cat# 2947; dil. 1:1000), actin (Sigma; cat# A2066, dil. 1:2000), GAPDH (Santa Cruz, cat# sc-47724; dil. 1:500).

### In vitro kinase assays

The effect of 10 µM palbociclib on a selected panel of kinases was assessed using the Reaction Biology assay platform (http://www.reactionbiology.com). Percent kinase activity remaining in treated samples relative to vehicle-treated controls was reported. All measurements were performed in duplicate.

### Patients survival analyses

Transcriptomic data from the Sidra-LUMC AC-ICAM colorectal cancer cohort (*n* = 303) were retrieved from cBioPortal (https://www.cbioportal.org/study/summary?id=coad_silu_2022). Normalized gene expression values were used for downstream analyses. To evaluate the prognostic impact of ERK5-related transcriptional programs, survival analyses were performed using top 700 genes modulated by ERK5-IN-1 in Ls174T cells, and multivariate Cox proportional hazards regression models to associate gene expression with overall survival. Based on the multivariate model, patients were stratified into molecular clusters by k-means clustering, and survival differences between groups were assessed using the Kaplan–Meier method with log-rank test. For validation, univariate Cox regression analyses were performed for each individual gene. Hazard ratios were calculated, and genes with significant associations (*p* < 0.05) were retained as candidates. All survival analyses and data visualizations were carried out in R (v4.3.0) with the *survival* and *survminer* packages. For multivariable Cox proportional hazards analysis, the clinical covariates consistently available for this cohort, namely patient age and gender, were incorporated. Age was modeled as a continuous variable, whereas gender was included as a categorical variable. Tumor stage and prior treatment history were not consistently available in the Sidra-LUMC AC-ICAM clinical annotation retrieved from cBioPortal, and could not be included in the analysis.

### Molecular docking

The docking experiments were performed using the software “Maestro”. The two crystal structures (inward-open and outward-open) used in the experiments were downloaded from the “RCSB Protein Data Bank”. Molecular docking was performed using the Glide program within the Maestro 13.4 Molecular Modeling Suite from Schrödinger, LLC, New York, NY. The X-ray crystal structures of CK2α and PI3Kδ in their DWG-in and DFG-in conformations were obtained from the RCSB PDB databank https://www.rcsb.org (PDB code 8BCY and 8S3R respectively). Protein structures were prepared according to the standard Maestro protein preparation workflow, which includes removal of bulk water molecules at over 5Å than the co-crystallised ligand, addition of hydrogen atoms, completion of missing loops, and assignment of force field parameters. Protonation states at pH 7.4 ± 2.0 were generated by Epik. Receptor grids were generated using the Receptor Grid Generation module using the centroid of the co-crystallized ligand as the center of the grid box; the box was set as a cube with a side length of 10 Å; van der Waals radii scaling factors for receptor atoms were set at 1.0 and partial charge cutoff at 0.25. Docked compounds were prepared using the LigPrep module of Maestro using the PLS-2004 force field to generate ionization states at pH 7.0 ± 2.0 and multiple conformers of each compound. All molecules were then docked using SP precision, and the results were visualized using PyMOL (www.pymol.org).

### Statistical analysis

Statistical differences between groups were assessed using the unpaired two-tailed Student’s t-test. A p-value < 0.05 was considered statistically significant. Data are presented as mean ± standard deviation (SD). Statistical significance was denoted as follows: * *p* < 0.05; ** *p* < 0.01; *** *p* < 0.001; **** *p* < 0.0001. All analyses were performed using GraphPad Prism version 10.

## Results

### Drug library screening

**Fig. 1:**
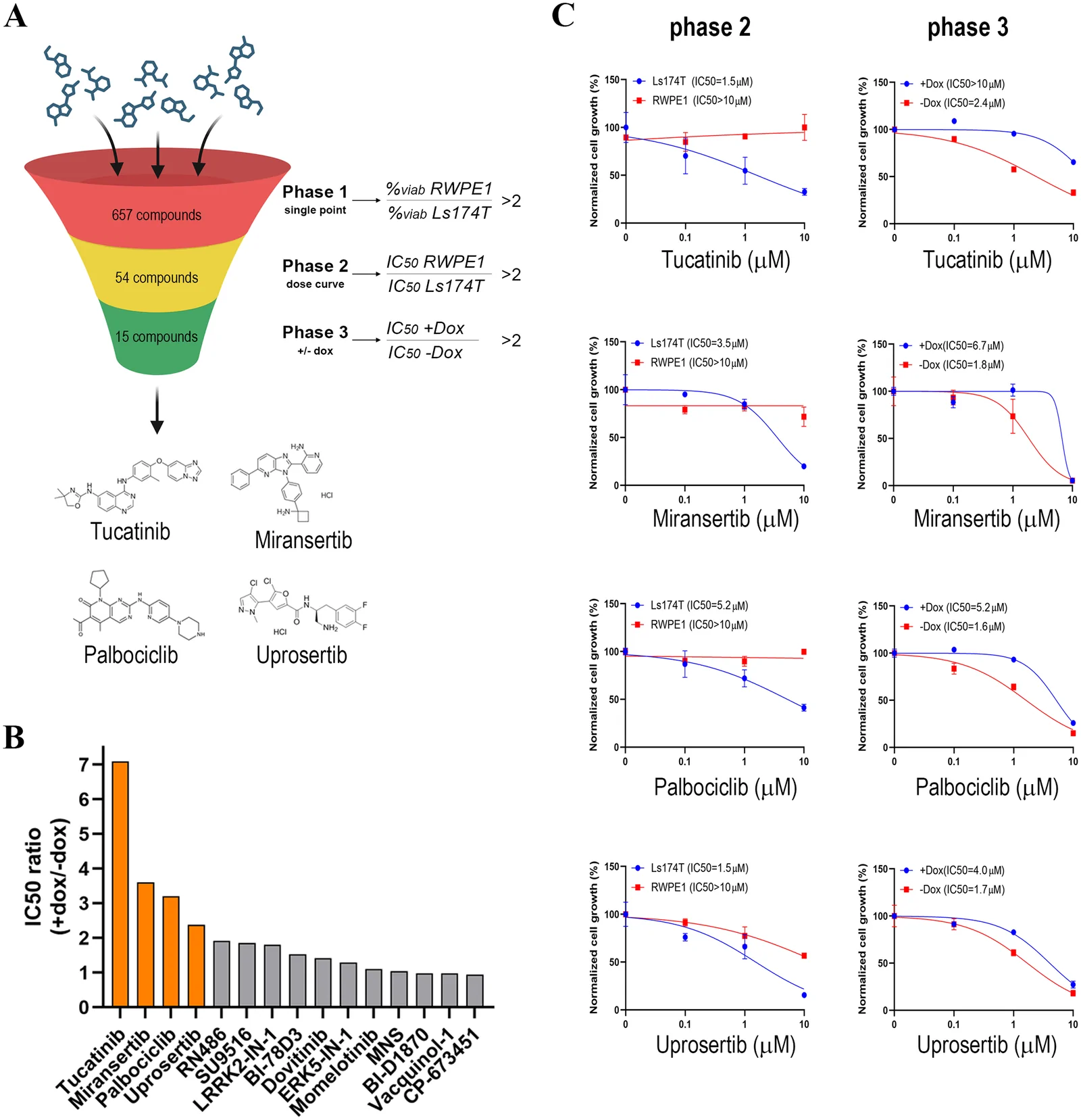
Identification of compounds selectively targeting aberrant β-catenin signaling. (**A**) Schematic overview of the multi-step screening strategy. (**B**) IC₅₀ ratios (+ dox/−dox) of the 15 inhibitors selected after phase 2 (see Supplementary Table 2); the four compounds passing phase 3 are indicated with orange bars. (**C**) Dose–response curves of Ls174T vs. RWPE1 cells (left, phase 2), and β-catenin-silenced (+ Dox) vs. control (− Dox) Ls174T cells (right, phase 3), treated with increasing concentrations of the indicated inhibitors. IC_50_ values are indicated above graphs. The curves were obtained by non-linear regression of normalized viability data

To identify compounds selectively interfering with aberrant β-catenin and KRAS signaling in CRC cells, we performed a multi-step screening of a chemical drug library (~ 660 compounds) using Ls174T cells harboring doxycycline-inducible shRNAs targeting β-catenin or KRAS [19] as a model (Fig. 1A).

Initial screening was conducted by treating Ls174T cells and a non-tumorigenic epithelial cell line, RWPE1, at a fixed drug concentration of 10 µM. RWPE1 cells served as a model for off-target toxicity, and the compound-selectivity threshold was set at a viability ratio (RWPE1/Ls174T) greater than 2. Based on this criterion, 54 compounds were selected for further investigation. In the second phase, dose-response curves were generated for the 54 selected compounds in Ls174T and RWPE1 cells. Compounds showing dose-dependent cytotoxicity in Ls174T cells, while sparing RWPE1 cells, were shortlisted for pathway specificity assessment in the third phase: 15 compounds (Fig. 1B and Supplementary Table 2) were tested in parental, β-catenin-silenced, and KRAS-silenced Ls174T cells, with the idea that shutting off oncogene expression (β-catenin or KRAS) would render the cells unresponsive to the action of a drug targeting that oncogene. Compounds showing ≥ 2-fold IC₅₀ increase upon doxycycline induction of target-specific shRNA were considered candidates for pathway-selective inhibition.

This stepwise approach led to the identification of four compounds endowed with selective activity against Ls174T cells and showing specific interference with active Wnt/β-catenin pathway (Fig. 1C). No compound showed analogous selectivity in KRAS-silenced cells (not shown). Interestingly, four additional compounds displayed selective cytotoxicity against Ls174T with no effects on RWPE1 cells, but their activity did not differ significantly between silenced (+dox) and control (-dox) conditions, suggesting that they may act through β-catenin-independent mechanisms that nonetheless compromise Ls174T cell viability (Supplementary Fig. 1A). Of note, two of these compounds, ERK5-IN-1 (XMD17-109) and LRRK2-IN-1, share significant structural similarity and common targets, such as ERK5 and BRD4 [28, 29]. These findings highlight candidate compounds that may either directly inhibit β-catenin signaling or act on alternative effectors synthetically lethal with β-catenin or KRAS activation, meriting further functional and mechanistic validation.

### Palbociclib characterization

Among the candidate compounds, palbociclib emerged as particularly promising due to its potent selective activity in Ls174T cells and its established clinical use, prompting its prioritization for in-depth characterization as a candidate modulator of β-catenin signaling in colorectal cancer. Palbociclib is a well-characterized CDK4/6 inhibitor, clinically approved for hormone receptor-positive breast cancer [30, 31]. CDK4/6 forms complexes with cyclin D1 to promote G1-S transition via phosphorylation of the retinoblastoma (Rb) protein [32]. However, its effects on β-catenin signaling are poorly characterized [33–35].

**Fig. 2:**
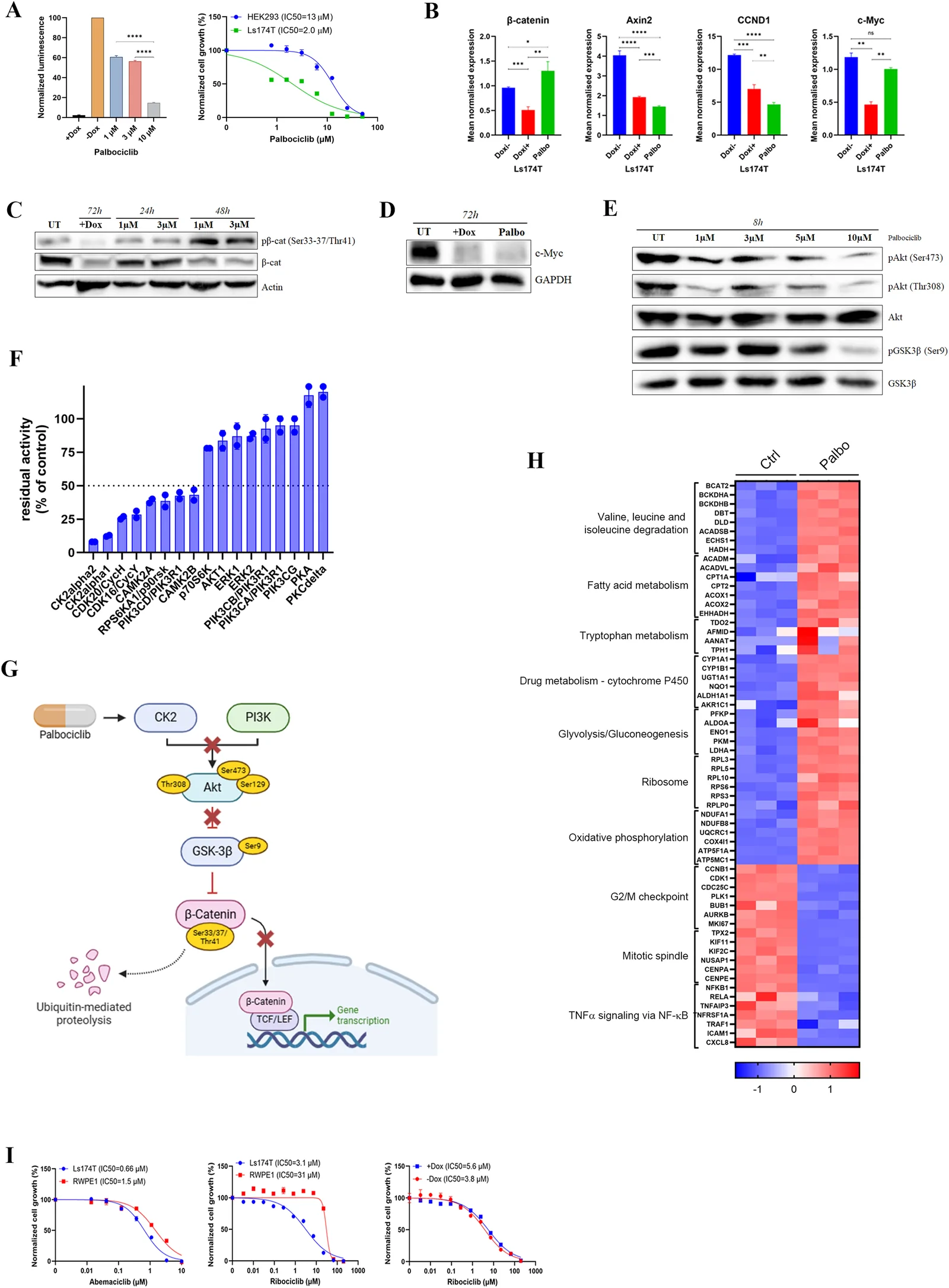
Characterization of palbociclib activity on the β-catenin pathway. (**A**) Luciferase activity from TOP-Flash reporter assay in Ls174T cells (left) and dose–response curves (right) obtained with palbociclib. (**B**) qPCR analysis of β-catenin and its target genes (AXIN2, CCND1, MYC) expression, comparing palbociclib and β-catenin silencing (+ Dox). (**C**) Western blot analysis of phosphorylated and total β-catenin, showing degradation at 48 h treatment. (**D**) c-Myc protein expression upon palbociclib treatment. (**E**) Western blot analysis of GSK3β and AKT phosphorylation. (**F**) In vitro kinase assay identifying kinases inhibited by palbociclib. (**G**) Schematic summary of the proposed mechanism of action. Palbociclib reactivates GSK3β by blocking CK2/PI3K/AKT signaling, thus forcing β-catenin degradation. (**H**) Heatmap of genes from enriched pathways identified by GSEA of palbociclib-treated versus untreated cells. (**I**) Effects of abemaciclib and ribociclib on Ls174T (± Dox) and RWPE1 cells, to test their specificity toward β-catenin signaling

We therefore explored the impact of palbociclib treatment on canonical Wnt signaling in CRC cells. We assessed β-catenin–dependent transcriptional activity using the TOPFlash reporter assay in Ls174T cells. Palbociclib treatment induced a clear, dose-dependent reduction in luciferase signal, consistent with direct interference with Wnt/β-catenin signaling. In parallel, palbociclib exerted a selective cytotoxic effect in Ls174T cells compared to non-CRC control cells (Fig. 2A). Palbociclib induced marked downregulation of two classical β-catenin target genes, AXIN2 and CCND1 (Fig. 2B), supporting the hypothesis that it interferes with Wnt pathway transcriptional output. The drug did not alter CTNNB1 transcription (Fig. 2B) but induced a significant reduction of β-catenin protein levels, as shown by western blotting, coupled with an increase in its phosphorylated form at Ser33/Ser37/Thr41, a modification associated with proteasomal degradation (Fig. 2C). These results suggest that palbociclib promotes β-catenin turnover. Interestingly, MYC transcript was not downregulated by palbociclib treatment (Fig. 2B), whereas c-Myc protein expression was strongly reduced (Fig. 2D), suggesting post-translational control, in line with previous observations [20, 36] (and our unpublished data). Myc is a key oncogenic driver in CRC, regulating cell growth, proliferation, metabolism and apoptosis [37]. Its strong downregulation likely mediates cell growth inhibition in Ls174T cells.

The classical mechanism of β-catenin degradation involves GSK3β, which phosphorylates β-catenin on specific residues leading to its ubiquitination. Notably, palbociclib treatment led to reduced phosphorylation of GSK3β on Ser9 (Fig. 2E), a modification that normally inhibits its kinase activity. This suggests that palbociclib may relieve the inhibitory block on GSK3β, thereby enhancing β-catenin degradation. Further supporting this mechanism, palbociclib also reduced phosphorylation of AKT (Fig. 2E). Because AKT inactivates GSK3β through Ser9 phosphorylation [38], its inhibition by palbociclib may reactivate GSK3β, forcing β-catenin turnover. To better understand the molecular players involved, we profiled the activity of palbociclib against a selected panel of recombinant kinases. This assay identified candidate kinases whose inhibition may correlate with the observed β-catenin destabilization (Fig. 2F). In particular, palbociclib strongly inhibited CK2, that has been involved in β-catenin regulation [39] and PI3Kδ which in turn activates AKT [40]. These findings are consistent with the data from KinomeScan profiling (https://lincs.hms.harvard.edu/kinomescan/), the protein-drug interaction database (proteomicsdb.org), and with chemoproteomics analyses performed in CRC cell lines [41, 42]. Interestingly, these studies also identified ERK5 as a palbociclib target; as noted above, two ERK5 inhibitors were among the top hits of our screening (Supplementary Fig. 1A), suggesting that ERK5 (also known as MAPK7) may be a candidate therapeutic target in CRC. Overall, these results suggest a mechanism of palbociclib action in CRC cells through inhibition of CK2 and PI3K isoforms, which translates into AKT inactivation and consequent release of GSK3β-dependent β-catenin degradation and cell growth arrest, as summarized schematically in Fig. 2G. Acute short-term treatment of mice with palbociclib reduced phospho-AKT levels and induced β-catenin phosphorylation (Ser33/Ser37/Thr41) in the tumors, confirming activation of the proposed mechanism (Supplementary Fig. 2A).

We then investigated the transcriptional effects of palbociclib treatment. Palbociclib induced upregulation of several metabolic pathways, suggesting that the cells are undergoing a metabolic reprogramming, possibly as a stress response. An enrichment of the ribosome pathway also emerged, suggesting sustained or adaptive protein synthesis activity. Concurrently, significant downregulation of proliferative and inflammatory pathways occurred, indicating that palbociclib effectively blocks cell cycle progression and dampens inflammatory signaling (Fig. 2H and Supplementary Fig. 2B). These data confirm that palbociclib not only arrests cell proliferation but likely causes metabolic stress, energy imbalance, and ultimately cellular toxicity.

To determine whether the observed effects were specific to palbociclib rather than a class effect of CDK4/6 inhibitors, we compared its activity with the structurally related inhibitors abemaciclib and ribociclib in Ls174T cells. This analysis confirmed the superior efficacy of palbociclib in this model. Indeed, abemaciclib showed poor selectivity for Ls174T over non-target cells, while ribociclib showed comparable effects in the presence or absence of doxycycline in Ls174T cells (Fig. 2I). These results were further confirmed by western blot analysis, which revealed no changes in β-catenin levels upon treatment (Supplementary Fig. 2C-D); however, all CDK4/6 inhibitors effectively reduced phospho-Rb levels, consistent with their expected mechanism of action on CDK4/6 (Supplementary Fig. 2E).

These results indicate that palbociclib activity on Wnt signaling is unique and is not shared by close analogues, also confirming that the mechanism of β-catenin inhibition by palbociclib is independent of its activity against CDKs. To elucidate this mode of action and explain the different behavior of palbociclib with respect to abemaciclib and ribociclib, we carried out in silico studies of the three inhibitors against the putative targets CK2α and PI3Kδ using the Maestro suite. In CK2, a DWG sequence replaces the common DFG motif in the activation segment of the kinase. When docked into the CK2α co-crystal structure in the DWG-in conformation (PDB code 8BCY) [43], palbociclib shows a high affinity binding pose (Supplementary Fig. 2F). Conversely, docking of abemaciclib and ribociclib on the same structure suggests lower affinity for CK2α (Supplementary Fig. 2G-H). Similarly, docking on PI3Kδ in the DFG-in conformation (PDB 8S3R) [44] suggests a higher target affinity of palbociclib compared with abemaciclib and ribociclib (Supplementary Fig. 2I-K). The docking pose of palbociclib shows extensive hydrogen-bond and hydrophobic interactions with residues delimiting the ATP-binding cavity of PI3Kδ. In contrast, the geometry of ribociclib and abemaciclib does not elaborate significantly into the ATP-binding pocket.

Finally, we explored palbociclib activity across various CRC cell lines with different oncogenic alterations (Supplementary Table 1). Palbociclib treatment increased Ser33/Ser37/Thr41 phosphorylation and reduced total β-catenin levels in HCT-116 cells, carrying a similar genetic makeup as Ls174T (Supplementary Fig. 2L). In contrast, SW480 and DLD-1 cells, harboring APC truncating mutations, did not show any reduction in β-catenin levels following palbociclib treatment (Supplementary Fig. 2M). These data reinforce the notion that palbociclib may promote β-catenin degradation through proteasomal targeting mediated by the destruction complex. However, in proliferation assays, CRC cell lines displayed variable sensitivity to palbociclib, not strictly correlated with their genetic background (Supplementary Fig. 2N). Interestingly, CDK4 expression inversely correlated with cytotoxic responses, with higher levels requiring elevated drug doses for comparable effects (Supplementary Fig. 2O). These results altogether suggest that palbociclib efficacy observed in Ls174T cells may reflect dual inhibition of CDK4/6 and β-catenin signaling.

We next evaluated whether other compounds identified in our screening also impacted β-catenin signaling. Interestingly, tucatinib and uprosertib also reduced GSK3β^Ser9^ phosphorylation and increased phospho-β-catenin^Ser33/Ser37/Thr41^ and degradation in Ls174T cells (Supplementary Fig. 3A-E). They contributed to β-catenin destabilization by modulating upstream kinase signaling: their actions likely converge on the GSK3β/β-catenin pathway by direct AKT inhibition (uprosertib [45, 46]) or via ERBB2 blockade (tucatinib).

Finally, we asked whether combining compounds could enhance their therapeutic potential. Given its established clinical approval [47, 48], tucatinib was prioritized as a partner of palbociclib in subsequent analyses. We first assessed the activity of tucatinib on β-catenin–dependent transcription using the TOPFlash reporter assay in Ls174T cells. Similar to palbociclib, tucatinib induced a dose-dependent inhibition of luciferase activity, consistent with interference with Wnt/β-catenin signaling, and displayed selective cytotoxicity in Ls174T cells compared with non-CRC controls (Supplementary Fig. 3F). When combined, the two drugs showed additive effects on viability and in TOPFlash assays, suggesting a common mode of action (Supplementary Fig. 3G-H and Supplementary Table 3). The addition of uprosertib to the palbociclib–tucatinib doublet improved efficacy but the effect was not consistently observed in other CRC lines (Supplementary Fig. 3I), suggesting that sensitivity may be context dependent.

To further explore combination strategies, palbociclib and tucatinib were each tested in combination with the top four compounds active in Ls174T cells independently of β-catenin expression (LRRK2-IN-1, ERK5-IN-1, BI-D1870 and Vacquinol-1). Drug concentrations that were tolerated by non-transformed RWPE1 cells were selected, to exclude excessive off-target toxicity, and evaluated in Ls174T cells (Supplementary Table 4). Several combinations demonstrated synergistic activity; among them, the one including ERK5-IN-1 was consistently the most synergic, while sparing normal cells (Supplementary Fig. 3J).

### Combination with KRAS inhibitors

**Fig. 3:**
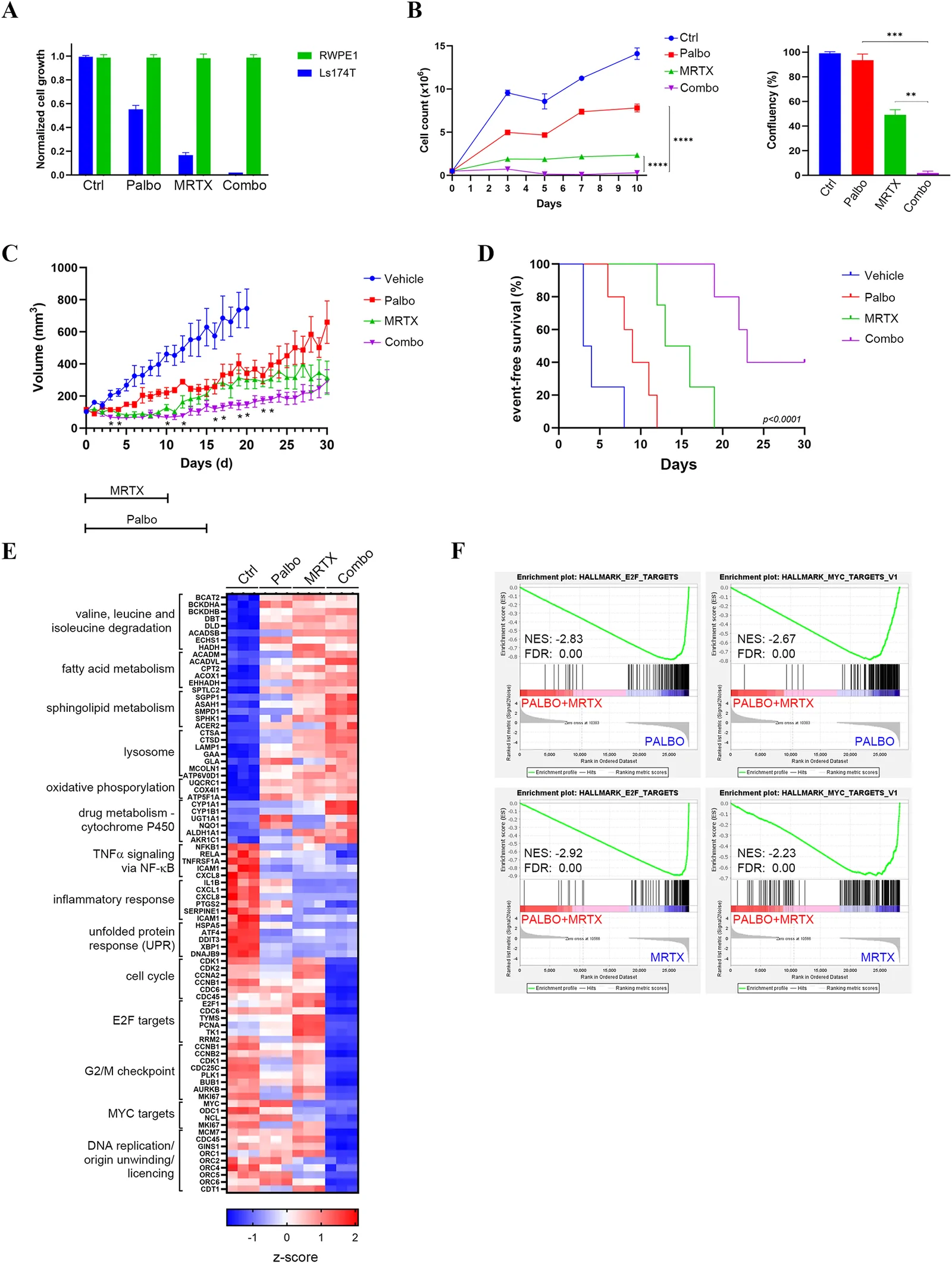
Characterization of the palbociclib + MRTX1133 combination. (**A**) Proliferation of Ls174T and RWPE1 cells treated with vehicle (Ctrl), palbociclib (Palbo, 3 µM), MRTX1133 (MRTX, 10 µM), or their combination (Combo). (**B**) Time course experiment in Ls174T cells over 10 days; cells were counted by Trypan blue (*left*) and confluence at day 10 was measured by ImageJ software on photographic images (*right*) (**C**) Tumor growth of Ls174T xenografts in nude mice treated daily with vehicle, palbociclib (75 mg/kg), MRTX1133 (10 mg/kg) or their combination; *, *p* < 0.05 (two-tailed t-test) combination versus both single treatments. Data are mean ± SD. (**D**) Kaplan–Meier estimation of event-free survival based on tumor doubling time; the p-value indicates curves comparison by log-rank test. (**E**) Heatmap of enriched gene expression signatures identified by GSEA in Ls174T cells. (**F**) GSEA plots comparing pathway enrichment in palbociclib + MRTX1133 versus single-agent treatments

As β-catenin and KRAS are two major drivers of CRC, we sought to investigate the therapeutic potential of combining palbociclib with recently developed KRAS inhibitors. Combination with the pan-KRAS inhibitor daraxonrasib (RMC-6236) [49] elicited synergistic effects in Ls174T and HCT-116 CRC cell lines, but not in non-tumoral RWPE1 cells (Supplementary Fig. 4A). We then tested mutation-selective KRAS inhibitors: in SW837 cells, carrying a KRAS G12C mutation, palbociclib combined with the KRAS G12C inhibitor sotorasib led to approximately 60% reduction in cell viability, indicating moderate anti-proliferative effects in this setting, but no synergy was observed (Supplementary Fig. 4B). Likely, the lack of synergism in this case is due to biallelic APC truncation in these cells, which interferes with β-catenin degradation. Next, MRTX1133, a KRAS G12D-specific inhibitor, was used in Ls174T cells harboring a KRAS G12D mutation. As expected, MRTX1133 alone induced a specific anti-proliferative effect (Supplementary Fig. 4C). Strikingly, combining MRTX1133 with palbociclib resulted in a potent and selective suppression of cell proliferation, indicating that simultaneous targeting of CDK4/6, β-catenin and KRAS G12D can yield enhanced anti-tumor efficacy (Fig. 3A-B and Supplementary Fig. 4D-E). In line with its selectivity profile, MRTX1133 showed no activity in CRC cell lines that carry different KRAS mutations (G12V and G13D) (Supplementary Fig. 4F). Notably, in vivo co-administration of palbociclib and MRTX1133 significantly delayed tumor regrowth after treatment discontinuation and improved survival, compared to monotherapies, emphasizing its translational relevance (Fig. 3C-D).

To characterize the transcriptional changes induced by MRTX1133 and its combination with palbociclib, we performed RNA sequencing and GSEA using KEGG and Hallmark gene sets (Fig. 3E and Supplementary Fig. 4G-J). Similarly to palbociclib, we observed a marked upregulation of multiple metabolic pathways in MRTX1133-treated samples, including valine, leucine and isoleucine degradation, fatty acid metabolism, sphingolipid metabolism, pentose and glucuronate interconversion, and lysosomal function. At the same time, downregulation of inflammatory and stress-related programs was observed, including TNFα signaling via NF-κB, inflammatory response, and the unfolded protein response. The combination of palbociclib and MRTX1133 further reinforced these transcriptional trends. Concomitantly, the dual treatment showed dramatic downregulation of proliferation-related programs, including cell cycle, E2F targets, G2M checkpoint, DNA replication, and MYC targets v1/v2, compared with either monotherapy (Fig. 3F and Supplementary Fig. 4G-J). In addition, significant enrichment of oxidative phosphorylation was observed in comparison to MRTX1133 alone. Finally, the combination further amplified repression of WNT-driven and proliferation-associated genes: in the palbociclib + MRTX versus palbociclib comparison (PM vs. P), several genes showed marked downregulation, including *MYC* (log2FC = − 1.76; padj = 0), *CCNE1* (log2FC = − 1.58; padj = 1.38E-54), *CCNE2* (log2FC = − 4.07; padj = 3.55E-33), *CDC25A* (log2FC = − 1.90; padj = 3.09E-83), *SNAI1* (log2FC = − 1.84; padj = 6.91E-13), *FOSL1* (log2FC = − 3.13; padj = 4.93E-73), and *BIRC5* (log2FC = − 4.47; padj = 5.14E-105).

These results indicate that combining palbociclib with MRTX1133 induces a broad transcriptional shift characterized by enhanced metabolic activity, suppression of proliferative and inflammatory programs, and activation of immune signaling, supporting synergistic inhibition of CDK4/6, β-catenin and KRAS G12D.

### Palbociclib and ERK5-IN-1 combination

**Fig. 4:**
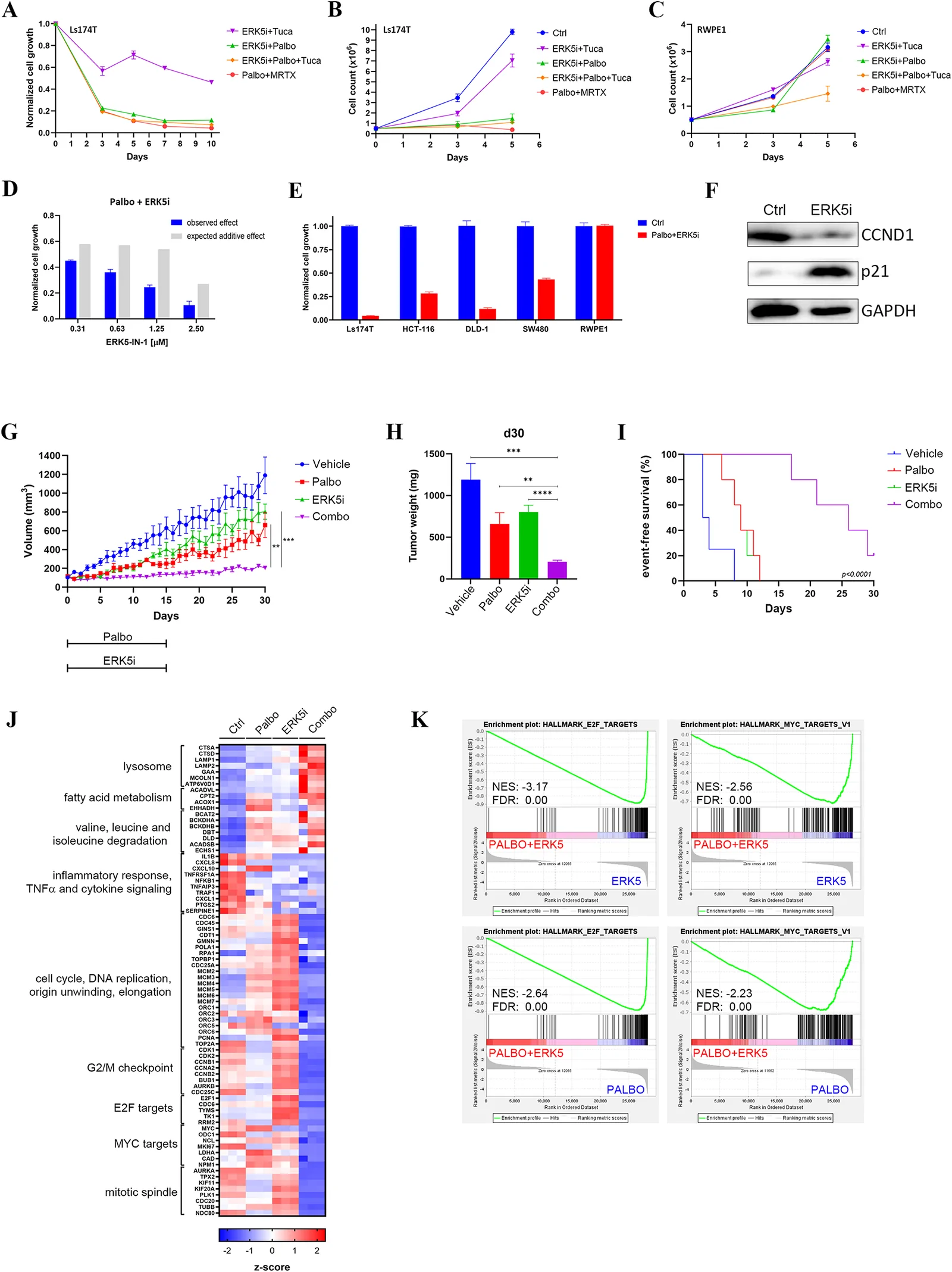
Characterization of the palbociclib + ERK5-IN-1 combination. (**A-C**) Time-course proliferation experiments of Ls174T and RWPE1 cells treated with vehicle, palbociclib (3 µM), ERK5-IN-1 (ERK5i, 1.25 µM), tucatinib (Tuca, 2.5 µM), MRTX1133 (10 µM) or their combinations. (**D**) Synergy of Palbo (5 µM) +ERK5i in Ls174T cells. (**E**) Cell viability of CRC cell lines treated with the combination palbociclib (10 µM) + ERK5-IN-1 (1.25 µM). (**F**) Western blot analysis of p21 and Cyclin D1 after ERK5-IN-1 treatment. (**G**) Tumor growth of Ls174T xenografts in nude mice treated daily with vehicle, palbociclib (75 mg/kg), ERK5-IN-1 (40 mg/kg), or the combination. (**H**) Tumor weight at day 30 post-treatment. (**I**) Kaplan–Meier analysis of event-free survival based on tumor doubling time (log-rank test). (**J**) Heatmap of enriched pathways identified by GSEA. (**K**) GSEA plots comparing palbociclib+ERK5-IN-1 versus single-agent treatments in Ls174T cells. Data are shown as mean ± SD. **, *p* < 0.01; ***, *p* < 0.001; ****, *p* < 0.0001 by one-way ANOVA with Tukey’s correction

Some of the most noteworthy drug combinations identified in our analysis involve ERK5-IN-1, a selective inhibitor of the ERK5 kinase. ERK5-IN-1 alone caused growth arrest of Ls174T cells with no cytotoxicity in control cells up to 10µM (Supplementary Fig. 5A). The compound was combined with tucatinib, palbociclib, or both, at its IC_50_ concentration, to identify the best combination, and compared with palbociclib+MRTX1133 combo. We found that palbociclib+ERK5-IN-1 treatment significantly impaired CRC cell growth with no significant toxicity in control cells, showing comparable efficacy to palbociclib+MRTX1133 (Fig. 4A-C). Combining palbociclib with the ERK5 inhibitor was strongly synergistic in Ls174T cells (Fig. 4D and Supplementary Fig. 5B) and when used on a panel of additional colorectal cancer cell lines resulted in a consistently strong anti-proliferative effect across all tested cells (Fig. 4E), suggesting that this may represent a promising pan-CRC treatment. To gain mechanistic insight into the observed effects, we analyzed the expression of key downstream targets. ERK5-IN-1 treatment led to a robust increase in p21 levels, a cyclin-dependent kinase inhibitor transcriptionally regulated by ERK5 signaling [50]. This was accompanied by a reduction in Cyclin D1 expression (Fig. 4F), suggesting that the combination effectively interferes with cell cycle progression through complementary mechanisms: CDK4/6 and β-catenin inhibition (via palbociclib) and ERK5 pathway blockade (via ERK5-IN-1).

Next, the therapeutic efficacy of palbociclib+ERK5-IN-1 combination was evaluated in vivo, compared with monotherapies. The combination elicited a rapid and pronounced anti-tumor effect, with tumor growth inhibition that was evident as early as day 6 of treatment (Fig. 4G). Mice were treated for 15 days and then followed up off treatment; on day 30 the mice were sacrificed: the combination group had significantly smaller tumors than either single treatment alone (Fig. 4H). Median event-free survival was 9 days versus 26 days for single treatments and the combination, respectively (Fig. 4I; *p* < 0.0001).

Finally, we investigated the transcriptional responses elicited by ERK5 inhibition and its combination with CDK4/6 and β-catenin blockade (Fig. 4J-K and Supplementary Fig. 5C-F). First, we observed strong downregulation of *KLF2* (log2FC= -2.16; padj.= 8.38*10E-51) and *KLF4* (log2FC= -2.04; padj.= 0.0), two known ERK5 targets, in ERK5-IN-1–treated samples, further confirming ERK5 inhibition [51, 52]. In general, ERK5-IN-1 unexpectedly induced significant upregulation of DNA replication and cell cycle–related pathways, while inflammatory signaling pathways were downregulated (Fig. 4J and Supplementary Fig. 5C). The addition of palbociclib to ERK5 inhibition markedly altered this transcriptional profile: upregulation of oxidative phosphorylation and several metabolic and biosynthetic programs was observed, including fatty acid and amino acid metabolism, accompanied by strong repression of the proliferation-associated signatures (Supplementary Fig. 5D). To dissect the specific contribution of palbociclib to the combination treatment, we compared transcriptomic profiles of combination-treated samples to those treated with ERK5-IN-1 alone. This analysis revealed upregulation of translational and mitochondrial processes, including translation initiation, ribosomal pathways, and oxidative phosphorylation (Supplementary Fig. 5F). Notably, we also observed enrichment of a mitochondria-associated gene set linked to electron transfer dysfunction, suggestive of enhanced or altered mitochondrial activity. At the same time, cell cycle and DNA replication–related programs remained strongly suppressed, as did Hallmark gene sets related to proliferation. In particular, E2F and MYC targets were significantly depleted in the combination compared with each single treatment (Fig. 4K). Together, these data indicate that ERK5 inhibition alone paradoxically upregulates the cell cycle machinery, but when combined with palbociclib, it results in robust anti-proliferative transcriptional reprogramming. This is characterized by coordinated suppression of DNA replication and mitotic progression, coupled with enhanced metabolic, translational, and immune-related signatures, suggesting synergistic effects of dual ERK5 and CDK4/6 inhibition in restraining tumor cell growth. Again, the combination showed sustained repression of WNT pathway and proliferative genes compared with palbociclib alone, such as *MYC* (log2FC = − 1.93; padj.= 3.75E-26), *CCNE1* (log2FC = − 1.22; padj.= 1.07E-06), *CCNE2* (log2FC = − 2.52; padj.= 1.59E-20), *CDC25A* (log2FC = − 3.00; padj.= 1.28E-57), *SNAI1* (log2FC = − 1.75; padj.= 5.22E-10), *FOSL1* (log2FC = − 1.31; padj.= 4.80E-08), and *BIRC5* (log2FC = − 4.62; padj.= 1.00E-66).

Next, as both combinations appeared to elicit mechanisms of metabolic remodeling in cells in response to proliferative arrest, we asked whether a cytostatic, rather than cytotoxic, response was in place. This is a critical distinction, as it might facilitate tumor dormancy and eventual recurrence, thereby complicating the long-term efficacy of the treatments. From RNA-seq data, *BCL2* gene was consistently downregulated by both combinations compared with single agents (palbociclib+ERK5i, log2FC range from − 1.48 to -1.86 in all comparisons; palbociclib+MRTX1133, log2FC range from − 0.66 to -1.12), which may in part account for the strong synergism. No other regulator of apoptosis was transcriptionally modulated by treatments. Hence, induction of apoptosis was evaluated upon combined treatments. The cells underwent marked apoptosis within 96 h of combined treatments (Supplementary Fig. 6), thus confirming that cell death follows growth arrest when palbociclib is combined with KRAS or ERK5 inhibitors.

**Fig. 5:**
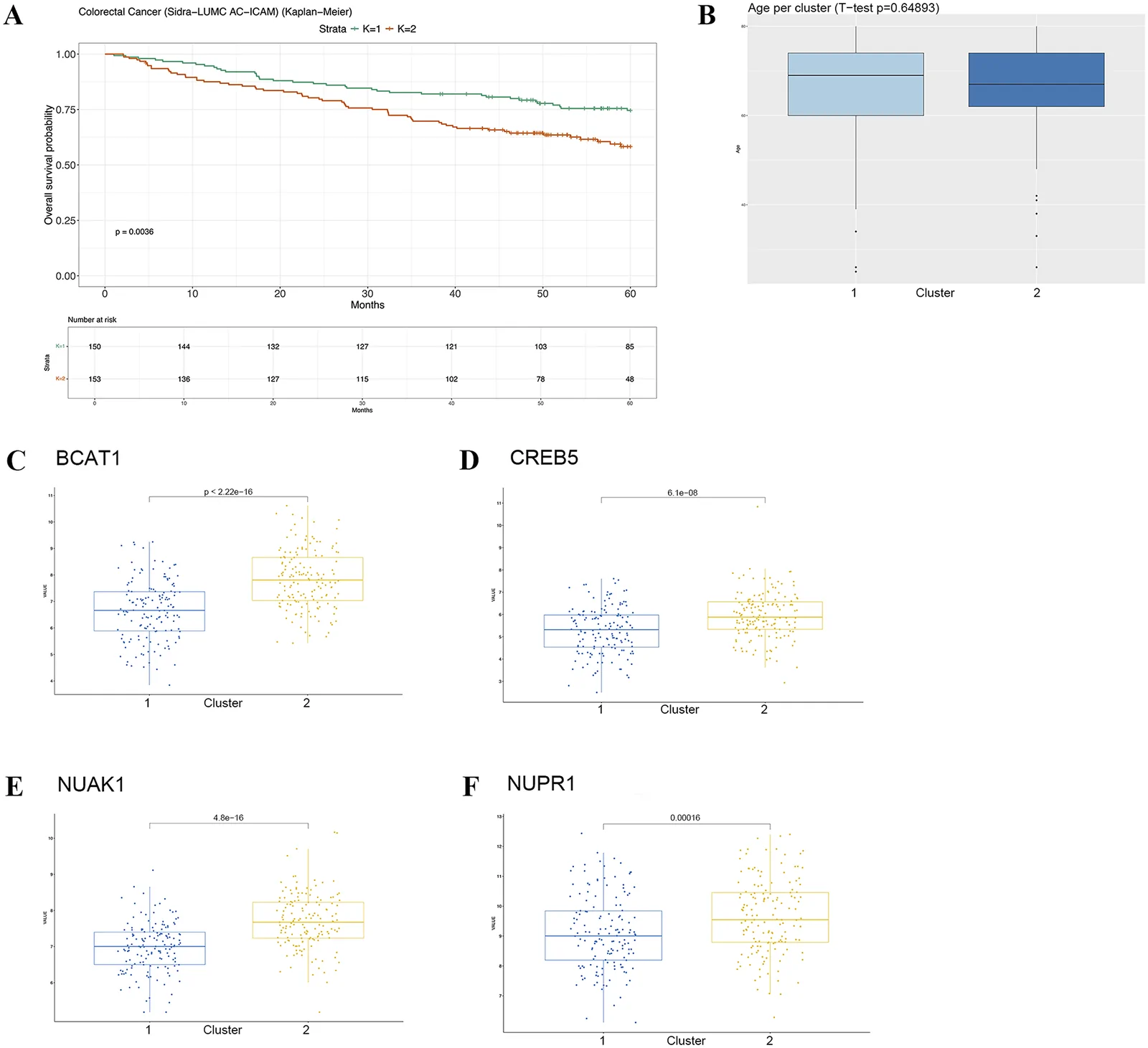
Bioinformatic analysis of ERK5-related transcriptional signatures. (**A-B**) Kaplan–Meier overall survival curve (**A**) and age distribution (**B**) of the two patient clusters identified by multivariate analysis. (**C-F**) Expression levels of BCAT1 (**C**), CREB5 (**D**), NUAK1 (**E**), and NUPR1 (**F**) across the two clusters

Finally, to assess the clinical relevance of our findings, we performed survival analysis using the Sidra-LUMC AC-ICAM colon cancer patients cohort (*n* = 303) [53] to evaluate the prognostic and therapeutic value of ERK5-related transcriptional signatures. Multivariate modeling associated gene expression with patient outcomes and stratified cases into two distinct clusters, which showed significantly different survival in Kaplan–Meier analyses, independent of age (Fig. 5A-B). In univariate analyses, we identified four functionally relevant genes, *BCAT1*, *CREB5*, *NUAK1* and *NUPR1*, that were strongly down-regulated by ERK5 inhibition in our dataset and significantly associated with poor prognosis, thus validating them as potential therapeutic targets (Fig. 5C-F and Supplementary Fig. 7). Importantly, the prognostic significance of *BCAT1*, *CREB5*, *NUAK1*, and *NUPR1* appears independent of known CRC mutations, as no mutation was significantly enriched in the patient clusters defined by the ERK5-related signature. A multivariable Cox proportional hazards regression model adjusted for patients’ age and gender was performed to further validate these findings. In this analysis, neither age nor gender was significantly associated with survival. Importantly, *CREB5* and *NUPR1* remained significantly associated with poorer overall survival after adjustment for age and gender (*p* = 0.013 and 0.0004, respectively). *BCAT1* retained a positive association with poorer outcome, although slightly above the conventional significance threshold (*p* = 0.058), whereas *NUAK1* did not remain independently significant in the adjusted model. This data suggests that *CREB5* and *NUPR1* act as prognostic markers across different genetic backgrounds, while *BCAT1* and *NUAK1* support the biological relevance of the ERK5-regulated transcriptional program but require further validation as independent prognostic biomarkers. Mechanistically, *CREB5* overexpression drives proliferation, migration and apoptosis resistance in CRC [54], while *NUPR1*, a stress-induced transcriptional regulator, fosters tumorigenesis and therapy resistance through PI3K/AKT/mTOR activation, autophagy modulation, and EMT [55–57]. *BCAT1* functions as an oncogene in several cancers by promoting proliferation, invasion, angiogenesis, and EMT via activation of the PI3K/AKT/mTOR pathway [58–60], and can be post-transcriptionally stabilized by HuR to enhance ERK5 signaling and therapy resistance [61]. *NUAK1* directly phosphorylates AKT at Ser473, thereby sustaining AKT activation and inhibiting GSK3β, a critical regulator of β-catenin [62]. Collectively, these results establish a mechanistic link between ERK5-associated networks, AKT/GSK3β/β-catenin signaling, and adverse clinical outcomes, emphasizing the therapeutic potential of targeting this axis in colorectal cancer.

These findings support the hypothesis that inhibition of CDK4/6, β-catenin and ERK5 may represent a broadly effective strategy in CRC models, targeting key nodes in proliferative and survival signaling while sparing non-tumorigenic cells. Intriguingly, both KRAS and ERK5 inhibition, when combined with palbociclib, caused profound suppression of E2F and MYC transcriptional programs, in line with their robust effects on tumor growth. Notably, the MYC targets signature was previously validated in colon carcinoma vs. normal mucosa comparisons (GSE20916 dataset from the GEO database [63]).

## Discussion

In this study, we applied a functional drug screening approach to identify selective vulnerabilities in CRC cells characterized by active β-catenin signaling and distinct KRAS mutations. Our results highlight the therapeutic potential of combining the CDK4/6 inhibitor palbociclib with agents targeting key oncogenic drivers, revealing novel combinatorial strategies for the treatment of molecularly defined CRC subtypes.

The initial screening identified candidate compounds with selective cytotoxicity in β-catenin-dependent CRC cells. Among these, palbociclib showed unexpected ability to induce β-catenin degradation via release of GSK3β-dependent β-catenin phosphorylation. In support of our findings, palbociclib was found to activate GSK3β in adrenal cancer cells by an unknown mechanism [35]. From our data we hypothesize that palbociclib modulates GSK3β activity through direct inhibition of CK2α and PI3Kδ. The latter has previously been postulated as a target of palbociclib [64]. Additional potential mediators of palbociclib action on β-catenin stability such as CaMKII or PKA, were ruled out (data not shown). Interestingly, in our study we detected robust phosphorylation of β-catenin at residues Ser33/Ser37/Thr41 despite loss of Ser45 in Ls174T and HCT-116 cells. According to the canonical model, phosphorylation at Ser45 by casein kinase I is required to prime subsequent phosphorylation at Ser33/Ser37/Thr41 by GSK3β. However, consistent with previous reports in colorectal cancer cells [65], we observed that phosphorylation at these downstream sites can occur independently of Ser45 status. It should be noted that the effect of palbociclib appeared to be dependent on APC status, since it was observed only in APC-wild-type cell lines. This mechanism limits its application to patients carrying non-mutated APC. It is important to note, however, that APC mutations occur in approximately 70–80% of CRCs, leaving 20–30% of patients with preserved APC function, in most cases with WNT pathway activation driven by alternative mechanisms [66]. This subset of patients may particularly benefit from therapeutic strategies exploiting β-catenin destabilization.

In an effort to further enhance therapeutic efficacy, we explored the therapeutic potential of combining palbociclib with other screening hits. The combination with ERK5-IN-1 elicited a robust anti-proliferative effect across multiple CRC lines carrying different oncogenic variants. The ERK5 pathway has emerged as a potential target to overcome therapeutic resistance and to modulate cell proliferation and inflammatory signaling in cancer cells [67–69], supporting its integration into combination regimens. Although ERK5 inhibitors have not yet progressed to clinical trials, accumulating preclinical evidence, including our results, highlights their substantial therapeutic promise, particularly when used in rational combinations. In vivo validation in xenograft models confirmed the therapeutic relevance of this combination: treatment with palbociclib+ERK5-IN-1 significantly delayed tumor growth in Ls174T xenograft models. Our data indicate that ERK5 acts in a pathway parallel to β-catenin rather than directly regulating its stability or transcription, supporting the rationale for combining ERK5 inhibitors with palbociclib, whose activity extends beyond CDK4/6 inhibition to include modulation of Wnt/β-catenin signaling in colorectal cancer. Compound ERK5-IN-1 has been described as a dual ERK5/BRD4 inhibitor [70]; in addition, binding of ERK5-IN-1 to ERK5, while blocking its kinase activity, can paradoxically activate its transcriptional activity [51]. Strong downregulation of *KLF2* and *KLF4* suggests that the compound is actually inhibiting ERK5 kinase in our cells [71]. On the other hand, upregulation of cell cycle-related genes hints to activation of downstream transcription. Therefore, it is difficult at present to conclusively discriminate from which of these mechanisms the effects observed in CRC cells derive.

In line with the well-known double dependency of CRC cells to Wnt and Ras pathways [19, 20], KRAS-G12D-specific inhibitor MRTX1133 exhibited strong and selective anti-proliferative activity in Ls174T cells harboring the KRAS G12D mutation and led to enhanced suppression of cell growth in vitro and in vivo in combination with palbociclib.

Transcriptomic profiling across our treatment conditions revealed a convergent transcriptional response driven by palbociclib, consistent with its dual activity as an inhibitor of CDK4/6 and β-catenin. As expected, palbociclib monotherapy resulted in a strong downregulation of cell cycle programs, confirming robust proliferative arrest. In parallel, we observed a consistent suppression of inflammatory and stress-related pathways, including TNFα signaling via NF-κB and the unfolded protein response, suggesting that palbociclib may also modulate the tumor inflammatory milieu. Beyond that, palbociclib induced the upregulation of multiple metabolic and biosynthetic programs. These findings are in line with a metabolic rewiring toward oxidative and lipid-based energy production and sustained protein synthesis, possibly reflecting a compensatory survival mechanism under proliferation blockade. The modulation of these processes may also be influenced by the inhibition of β-catenin signaling, which is known to regulate metabolic gene expression and stem-like features in various tumor contexts.

When combined with KRAS inhibition (MRTX1133), palbociclib further amplified these effects. The dual treatment enhanced mitochondrial metabolism and lysosomal activity while deepening repression of cell cycle and inflammatory programs. Notably, the combination induced type I interferon signaling and stress-related immune responses, suggesting that CDK4/6 and β-catenin inhibition can enhance the immunogenicity of RAS-driven tumors and promote a more inflammatory microenvironment, potentially contributing to therapeutic synergy. A similar reprogramming was observed with the combination of palbociclib and an ERK5 inhibitor. Interestingly, while ERK5 inhibition alone paradoxically upregulated DNA replication and cell cycle–associated pathways, the addition of palbociclib restored and amplified anti-proliferative control and induced biosynthetic and mitochondrial gene sets. The coordinated suppression of MYC, E2F, and mitotic programs, together with the activation of oxidative phosphorylation, translation, and lysosomal function, further supports the idea that dual CDK4/6 and β-catenin inhibition drives tumor cells into a metabolically active yet non-proliferative state. Interestingly, a MYC module was commonly suppressed by both combinations, implying that the two treatments converge mechanistically on a common outcome. As MYC is a master regulator of cell growth, metabolism and proliferation, and a driver oncogene in CRC, we are likely hitting a key vulnerability. Overall, the transcriptomic profile shows that cells primarily engage metabolic adaptation programs in response to proliferative arrest, yet they undergo significant apoptosis upon combined treatments. This is mirrored by robust tumor growth inhibition in mice, supporting the therapeutic relevance of these transcriptional programs.

Altogether, our findings highlight the broad impact of palbociclib, which extends beyond CDK4/6 inhibition to include β-catenin–mediated pathways. This dual activity likely contributes to both the anti-proliferative and metabolic/inflammatory rewiring observed in our models. In combination with targeted inhibitors such as MRTX1133 or ERK5-IN-1, palbociclib acts as a central modulator that enhances tumor cell differentiation, suppresses proliferation, and promotes metabolic and immunogenic vulnerabilities, providing a strong rationale for its inclusion in combinatorial therapeutic strategies.

Importantly, survival analysis of the Sidra-LUMC AC-ICAM CRC cohort demonstrated that ERK5-related transcriptional signatures, including *BCAT1*, *CREB5*, *NUAK1*, and *NUPR1*, are consistently associated with poor prognosis. These results provide clinical validation of our experimental findings, establishing a link between ERK5 and AKT/GSK3β/β-catenin signaling and adverse outcomes in CRC patients, and further underscore the translational potential of targeting this axis through palbociclib-based combinations.

Finally, while the present work focused on defining the mechanistic basis and therapeutic potential of palbociclib-based combinations, future studies will be required to assess the development of acquired resistance to these regimens and to evaluate their efficacy in more advanced preclinical systems. In particular, long-term drug exposure models, as well as orthotopic and patient-derived xenograft models, will be instrumental to identify potential bypass mechanisms and to validate the translational relevance of these combinations in a context that more faithfully reflects human CRC. Potential mechanisms of resistance to palbociclib-based therapeutics may include loss or inactivation of RB pathway, CDK4/6 amplification or mutation, loss of CDK inhibitors, as well as reactivation of AP-1 signaling or PI3K/AKT pathway [72]. The latter may be investigated for resistance in the context of palbociclib-mediated β-catenin inhibition. On the other hand, combinations with KRAS and ERK5 inhibitors may also become ineffective due to mechanisms targeting these pathways, such as secondary KRAS mutations or upstream reactivation of wild-type RAS isoforms, or BRAF mutations.

## Conclusions

Our findings provide a compelling rationale for the development of tailored combination therapies involving palbociclib and simultaneously targeting CDK4/6, β-catenin, and either ERK5 or KRAS, in CRC. More broadly, our study demonstrates the power of a phenotype-driven screening approach to uncover rational combination therapies that exploit oncogene-specific vulnerabilities in CRC. The integration of functional screening with molecular characterization enabled the identification of effective and selective drug combinations, particularly in genetically stratified models. These approaches may ultimately improve clinical outcomes for patients with specific molecular subtypes of colorectal cancer. The effects of palbociclib on β-catenin degradation may potentially extend to other WNT-driven cancers beyond CRC. Whether palbociclib-based combinations will be effective in these settings, remains to be investigated. Finally, despite promising results, several limitations remain, including the small number of cell lines tested and the need for validation in more physiologically relevant models (such as patient-derived xenografts and organoids, or orthotopic systems) as well as more detailed molecular dynamics simulations to assess conformational flexibility and sustained drug occupancy of the drug within the CK2α and PI3Kδ binding pockets [73]. Also, extended time-course pharmacodynamic studies in mice are planned to definitively demonstrate in vivo degradation of total β-catenin pool. In addition, while showing strong preclinical activity, ERK5 inhibitors have not yet reached clinical development; therefore, their therapeutic potential should be interpreted with caution, as their pharmacokinetic and toxicity profiles in human subjects remain undefined. Finally, not all clinical covariables could be used for multivariate analysis due to incomplete annotation of the original public datasets.

## Supplementary Information

Below is the link to the electronic supplementary material.

**Supplementary Material 1**: Supplementary Fig. 1. Compounds interfering with Ls174T cell viability through β-catenin–independent mechanisms. (A) Dose–response curves of Ls174T vs. RWPE1 cells (left, phase 2), and β-catenin-silenced (+ Dox) vs. control (− Dox) Ls174T cells (right, phase 3), treated with increasing concentrations of the indicated inhibitors. The chemical structures of these compounds are shown on the right

**Supplementary Material 2**: Supplementary Fig. 2. Characterization of palbociclib effects. (A) In vivo modulation of β-catenin and AKT phosphorylation: acute treatment of mice induced significant decrease of phospho-AKT (Thr308) and upregulation of phospho-β-catenin (Ser33/37/Thr41), indicating activation of the degradation process, in palbociclib-treated mice. Total β-catenin was still unchanged, as degradation is expected to occur at later time points that were not captured at the time of sacrifice. One lane containing the UT sample loaded at a lower amount was removed from the image. (B) GSEA plots of palbociclib-treated versus untreated Ls174T cells comparisons. NES, normalized enrichment score; FDR, false discovery rate q-value. (C-D) Total β-catenin expression following treatment of Ls174T cells with abemaciclib (C) or ribociclib (D). The effect on cells treated with doxycycline (+ Dox) is shown for control. (E) Western blot analysis assessing the efficacy of CDK4/6 inhibitors on Rb phosphorylation. (F-H) Docking poses of palbociclib (F), ribociclib (G), and abemaciclib (H), within the binding site of CK2α (PDB code 8BCY). Hydrogen bonds are rendered as dotted lines, and key active site residues are labeled. Palbociclib binds CK2 with the piperazine group oriented towards the solvent and forming hydrogen (H) bonds with Arg43 and Asp120, the aminopyridino group forming H bonds with Val116 of the hinge and π-stacking interactions with His115, and the terminal acetyl group extending toward the Lys68–Glu81 salt bridge and forming a water-mediated hydrogen bond with Asp175 of the DWG motif. Ribociclib replicates the elaboration towards the solvent but does not form any H bond with Arg43, does not show any significant interactions with the inner part of the pocket and orients the amide methyl groups unfavorably toward the polar environment of the salt bridge, whereas abemaciclib docks outside the binding pocket. (I-K) Docking poses of palbociclib (I), ribociclib (J), and abemaciclib (K), within the binding site of PI3Kδ (PDB code 8S3R). Hydrogen bonds are rendered as dotted lines, and key active site residues are labeled. Palbociclib docks with the piperazine group oriented towards the solvent forming an H bond with Asp832, the aminopyrazine group forming hydrogen bonds with the backbone of Val828 and π-stacking interactions with Trp760, and the polar acetyl-pyrazinone moiety extending toward the DFG motif, forming a hydrogen bond with the phenolic group of Tyr813 and a water-mediated hydrogen bond with Asp911. Finally, the cyclopentyl group forms hydrophobic contacts with the side chain of Met752. Conversely, the geometry of ribocilib, with bulky groups in ortho positions, does not allow the elaboration of the purine moiety inside the active pocket and only affords low-affinity poses, with the dimethylamide and cyclopentyl groups oriented toward the solvent. Similarly, the sterically demanding abemaciclib positions its lipophilic isopropyl group toward the solvent. (L-M) Western blot analysis of β-catenin degradation following palbociclib treatment in HCT-116 (L), SW480, and DLD-1 (M) cells. (N) IC50 values of palbociclib across different CRC cell lines. (O) Western blot analysis of CDK4/6 expression levels in different CRC cell lines

**Supplementary Material 3**: Supplementary Fig. 3. Evaluation of combination treatments. (A-E) Western blot analysis of β-catenin degradation and upstream modulators, following treatment with tucatinib (A-C) or uprosertib (D-E). While reducing RPS6 phosphorylation, indicating inhibition of AKT/mTORC1 downstream signaling, uprosertib caused a paradoxical increase of AKT phosphorylation, consistent with a feedback compensatory mechanism upon direct enzyme inhibition. (F) Luciferase activity from TOP-Flash reporter assay in Ls174T cells (left) and dose–response curves (right) obtained with tucatinib. (G–H) Effects of palbociclib/tucatinib combinations on cell viability (G) and β-catenin transcriptional activity by TOP-Flash assay (H); in panel G, "*observed*" refers to experimental viability measures, while "*expected*" refers to predicted additive effects of the combination. (I) Summary of triple drug combination tested and its effects in Ls174T and other CRC cells. Drug concentrations were as follows: palbociclib 3 µM, tucatinib 0.63 µM, uprosertib 0.63 µM. Data are normalized to untreated control cells. (J) Summary of experimental drug combinations (1 µM + 1 µM) tested in Ls174T and RWPE1 cells. BI-D1870 was used at 10 µM. Grey bars indicate the expected residual Ls174T cell viability assuming drug independence (i.e., additive effect). Several combinations synergistically reduced viability in Ls174T cells without affecting control RWPE1 cells; Bliss values are also reported in the graph

**Supplementary Material 4**: Supplementary Fig. 4. Evaluation of toxicity and efficacy of KRAS inhibitors combined with palbociclib. (A) Activity of palbociclib (2.5 µM) combined with the pan-RAS inhibitor daraxonrasib (5 nM) on the proliferation of the indicated cells. Expected effect indicates the additive effect of the combination as predicted by Bliss independence model. Bliss scores are reported in the graph. (B) Effects of palbociclib + sotorasib combination on cell viability. (C) Dose–response MTS assay of Ls174T and RWPE1 cells treated with MRTX1133. (D) Time-course proliferation of Ls174T cells treated with vehicle alone (UT), palbociclib (3 µM), MRTX1133 (10 µM), or the combination. (E) Growth of Ls174T and RWPE1 cells treated with the indicated combinations for 10 days; ns, non-significant by t-test; ***, p < 0.001; ****, p < 0.0001. (F) Cell viability of CRC cell lines harboring non-G12D KRAS mutations, treated with palbociclib + MRTX1133. (G-J) GSEA analysis of Ls174T cells: MRTX1133 vs. untreated (G), palbociclib+MRTX1133 combination vs. untreated (H), combination vs. palbociclib (I), combination vs. MRTX1133 (J); analyses were performed using KEGG and Hallmark gene sets

**Supplementary Material 5**: Supplementary Fig. 5. Gene expression profiling of the palbociclib + ERK5-IN-1 combination. (A) Dose-response MTS assay of Ls174T and RWPE1 cells treated with ERK5-IN-1. IC50s are shown below the graph. (B) Heatmap of Bliss synergy scores obtained by combinations of palbociclib with ERK5-IN-1 in Ls174T cells. (C-F) GSEA diagrams of Ls174T cells: ERK5-IN-1 vs. untreated (C), palbociclib+ERK5-IN-1 vs. untreated (D), palbociclib+ERK5-IN-1 vs. palbociclib (E), palbociclib+ERK5-IN-1 vs. ERK5-IN-1 (F), using KEGG and Hallmark gene sets

**Supplementary Material 6**: Supplementary Fig. 6. Induction of apoptosis by combinations. Ls174T cells were treated with vehicle (Control), Palbociclib (3 µM), Palbociclib+MRTX1133 (10 µM), or Palbociclib+ERK5-IN-1 (1.25 µM) for 72 (A, C) or 96 h (B, D). Apoptosis was measured by Annexin V/7-AAD staining. The mean ± SD of biological triplicate values is reported in panels A and B. Early apoptotic (Annexin V positive, 7-AAD negative), late apoptotic (Annexin V and 7-AAD positive) and live cells (double negative) are reported. Dot plots from one representative experiment are shown in C and D

**Supplementary Material 7**: Supplementary Fig. 7. Expression of ERK5-associated genes following ERK5 inhibition Expression of *BCAT1*, *CREB5*, *NUAK1* and *NUPR1* in Ls174T cells treated with ERK5-IN-1 or DMSO vehicle (Ctrl)

Supplementary Material 8

Supplementary Material 9

Supplementary Material 10

Supplementary Material 11

Supplementary Material 12

## Data Availability

The datasets and the materials used and/or analysed during the current study are available from the corresponding author on reasonable request. Raw NGS data will be deposited in NCBI’s Sequence Read Archive.
