# Supplementary material for: Palbociclib targets β-catenin for degradation and synergizes with KRAS or ERK5 inhibition in colorectal cancer preclinical models
Source: J Transl Med. 2026 Jun 19;24:1132. doi: 10.1186/s12967-026-08420-7 (PMC13540963; doi:10.1186/s12967-026-08420-7)

Figure 2C

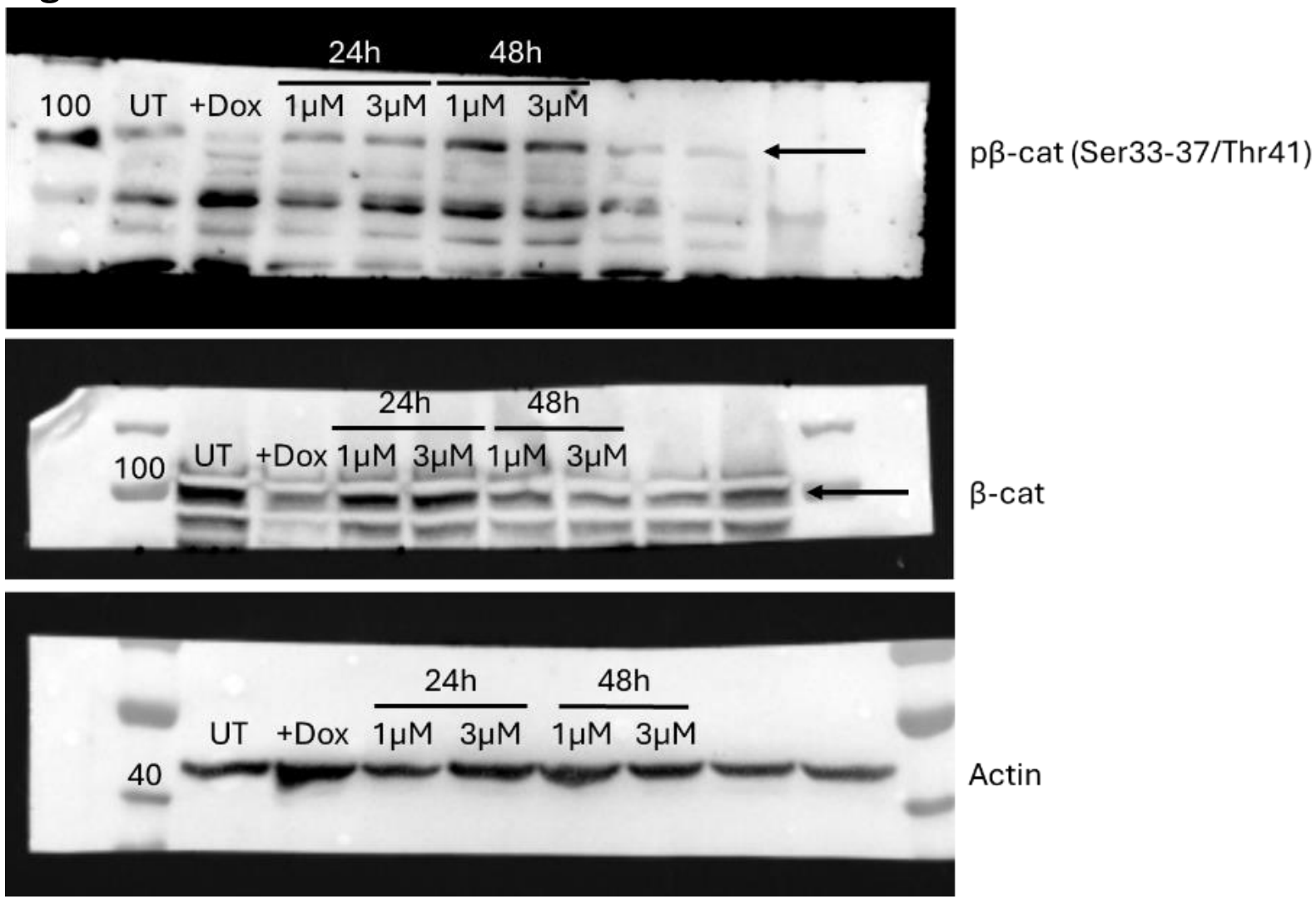

Figure 2D

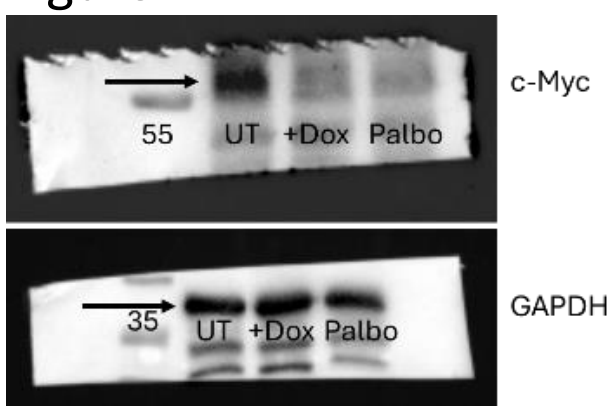

Figure 2E

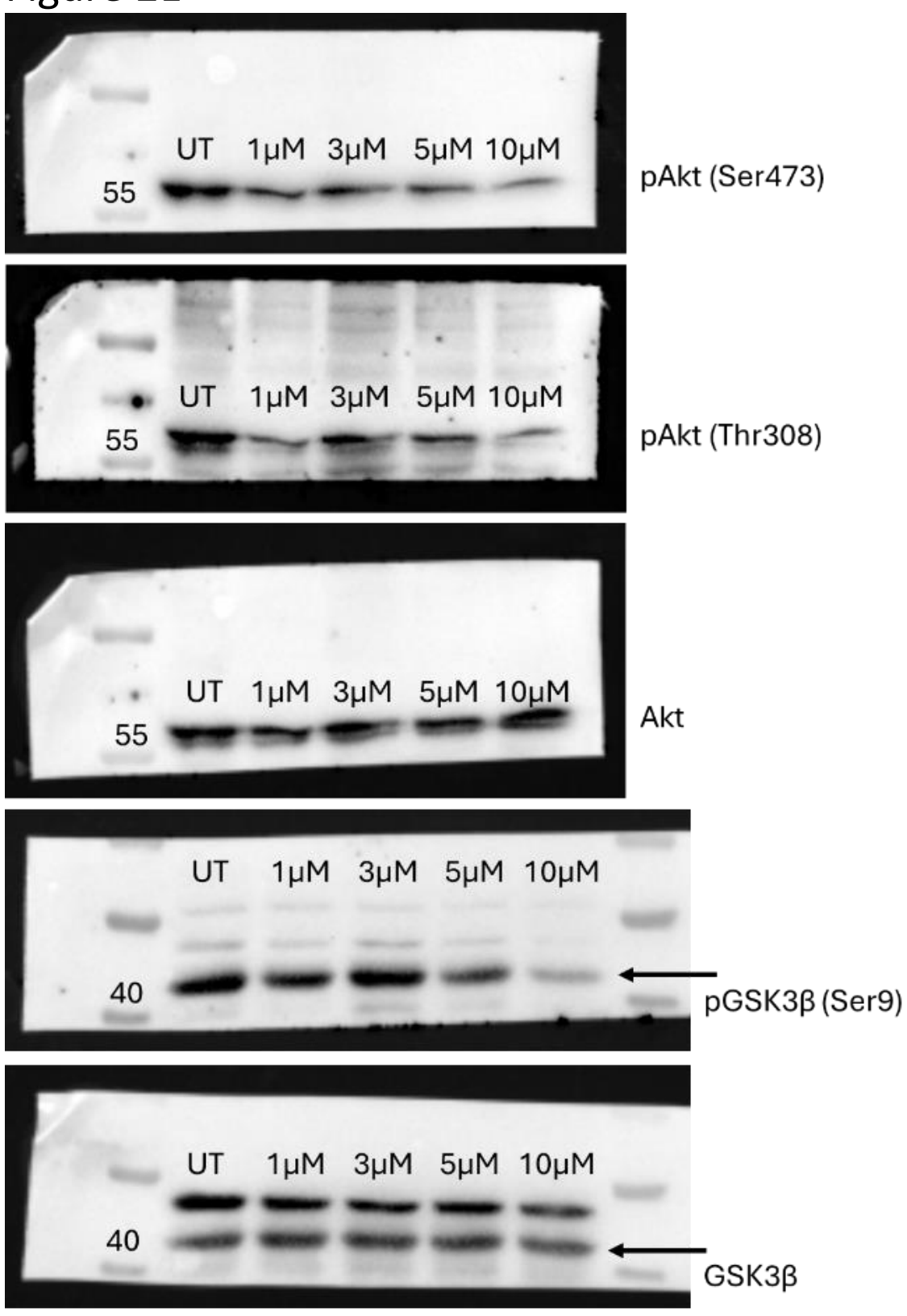

Figure 4F

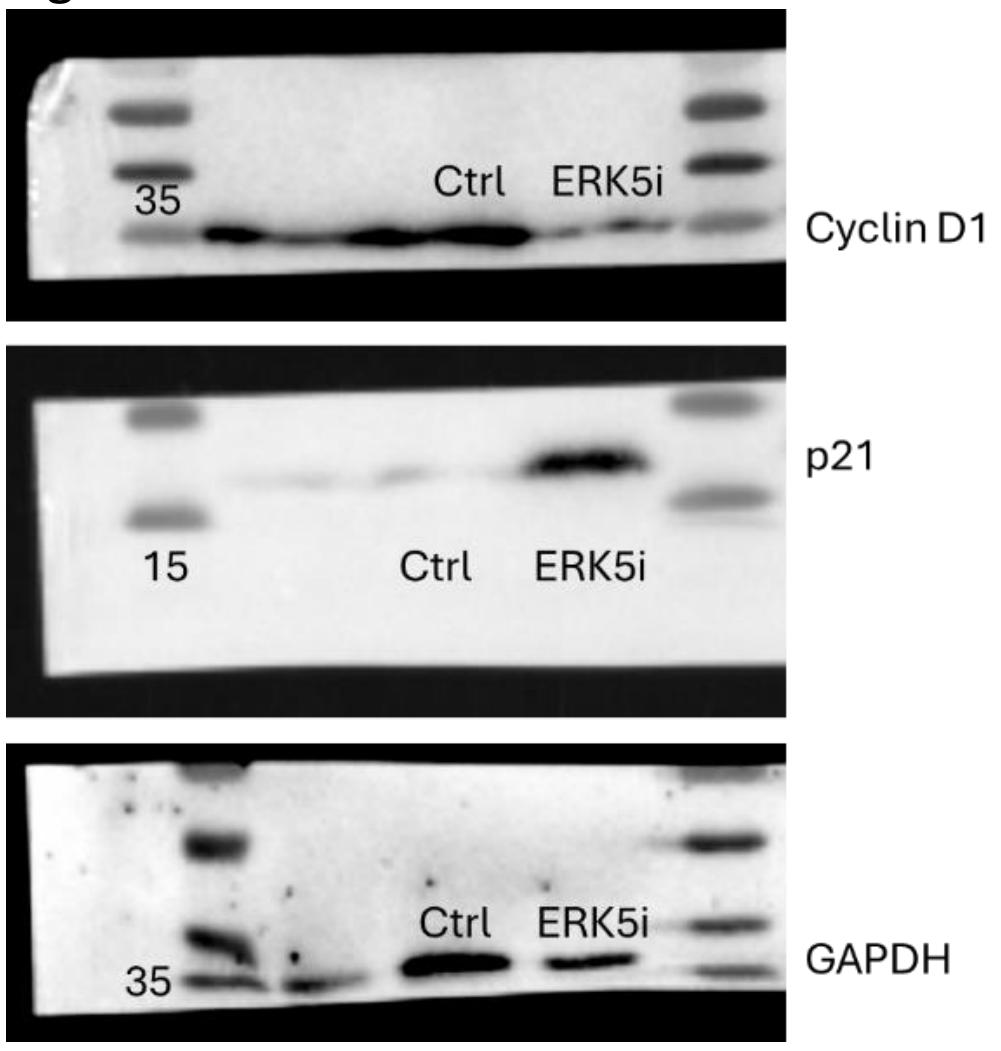

Sup. Figure 2A

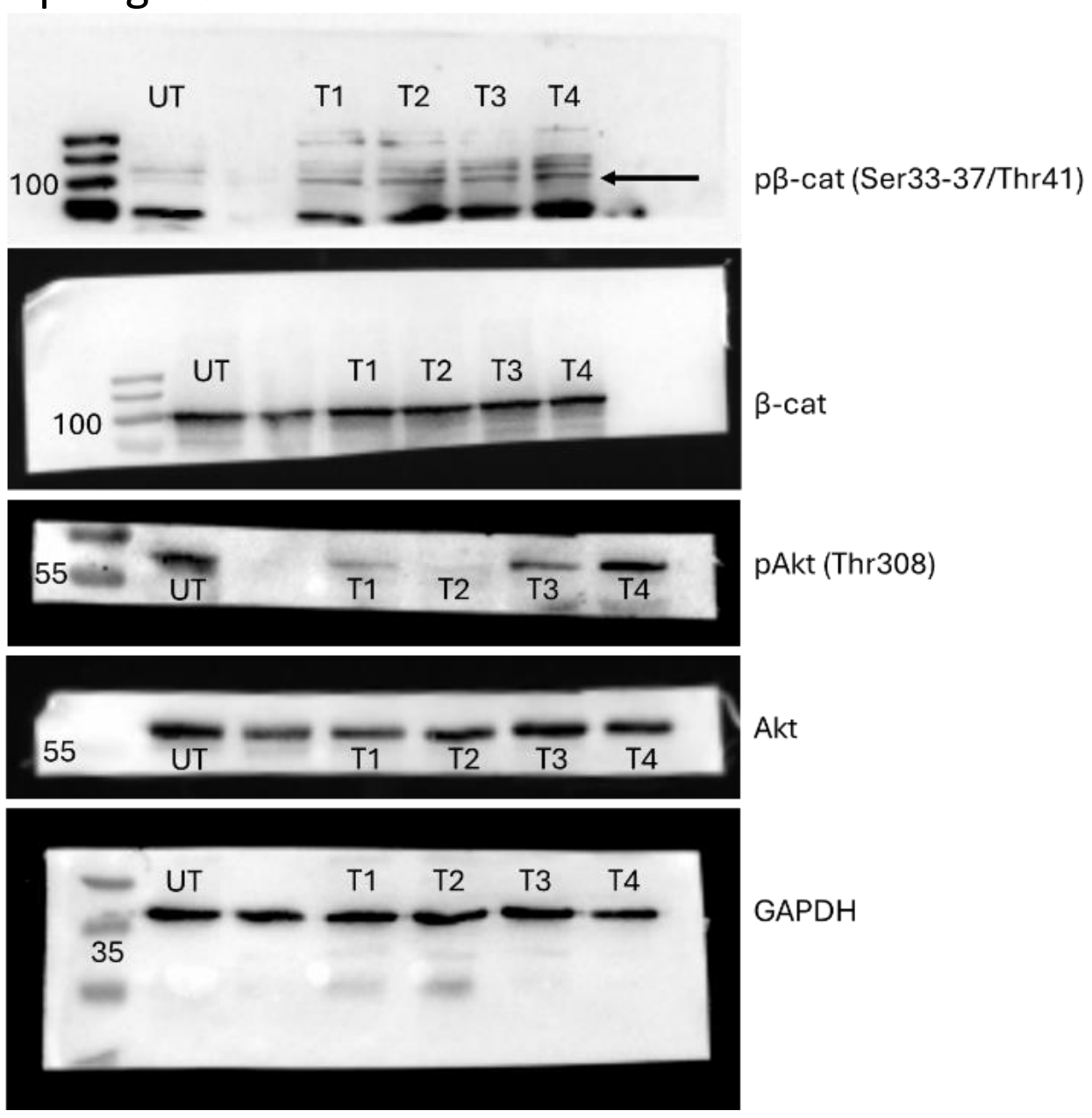

Sup. Figure 2C

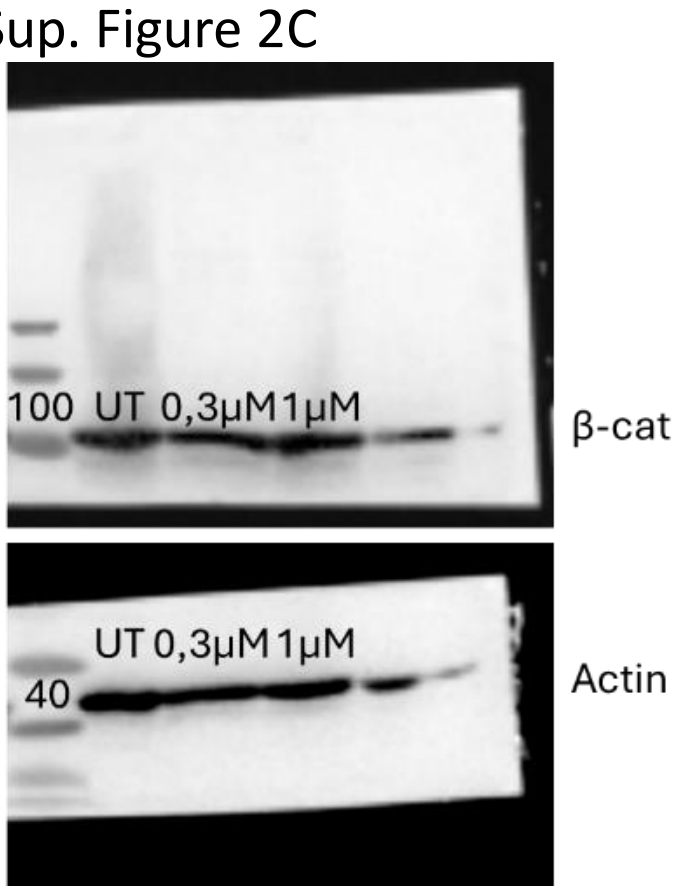

Sup. Figure 2D

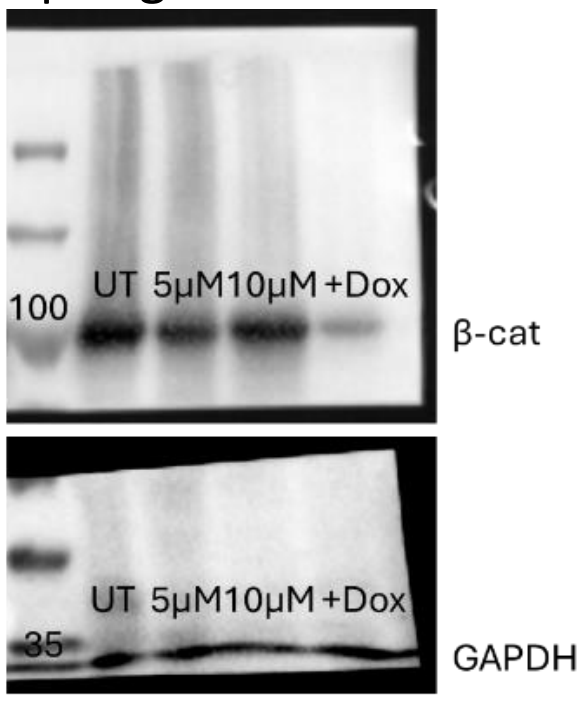

Sup. Figure 2E

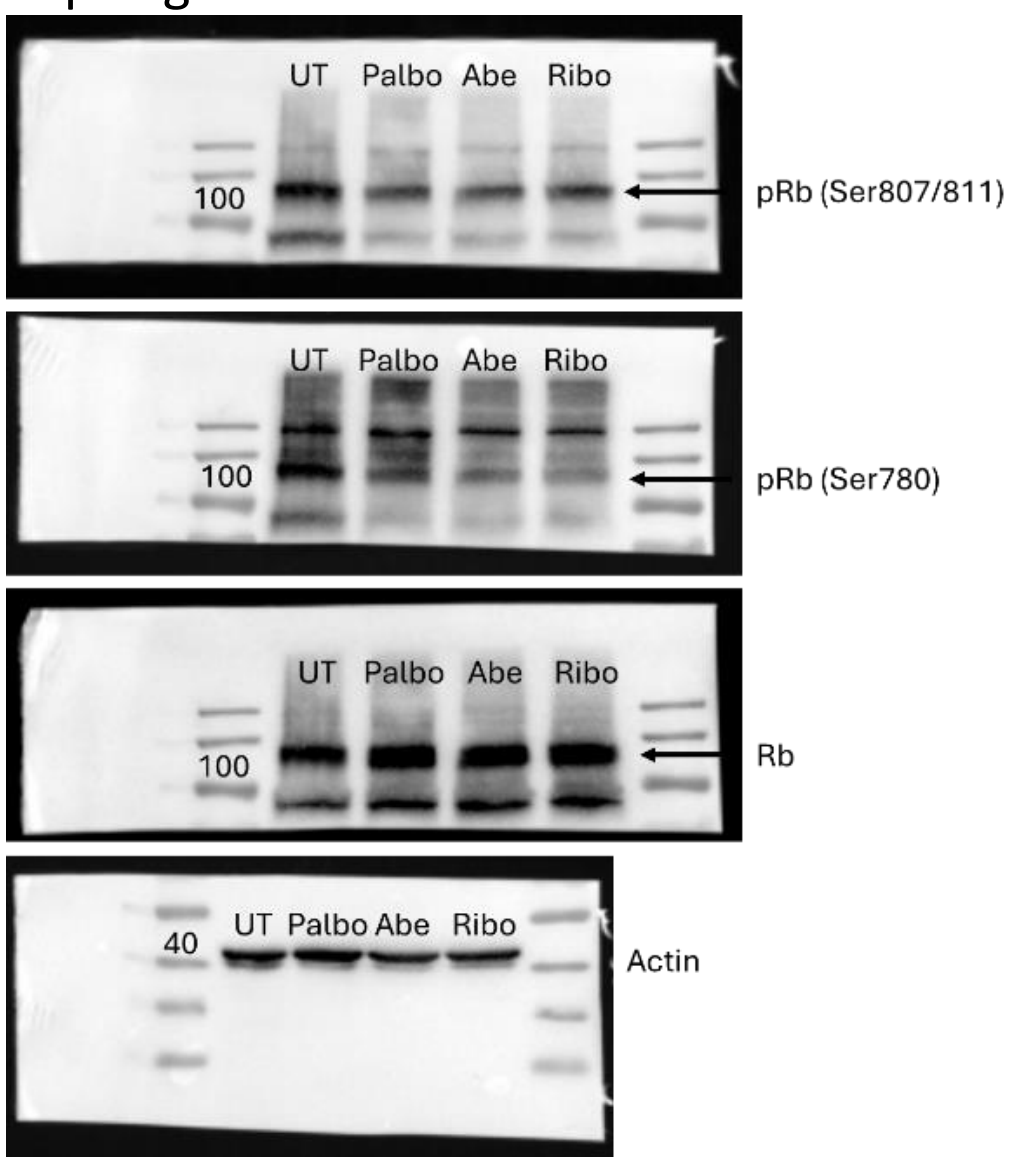

Sup. Figure 2L

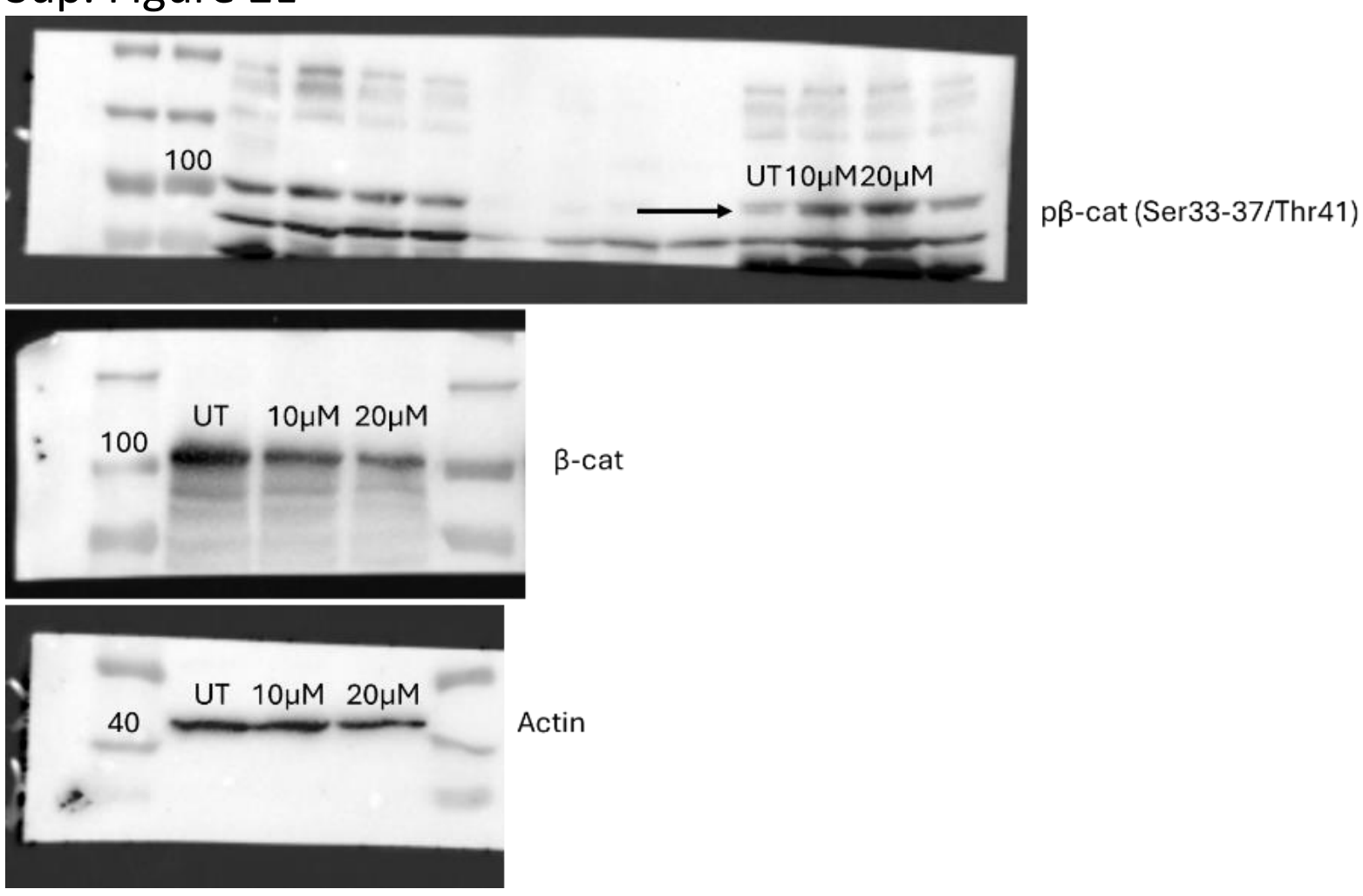

Sup. Figure 2M

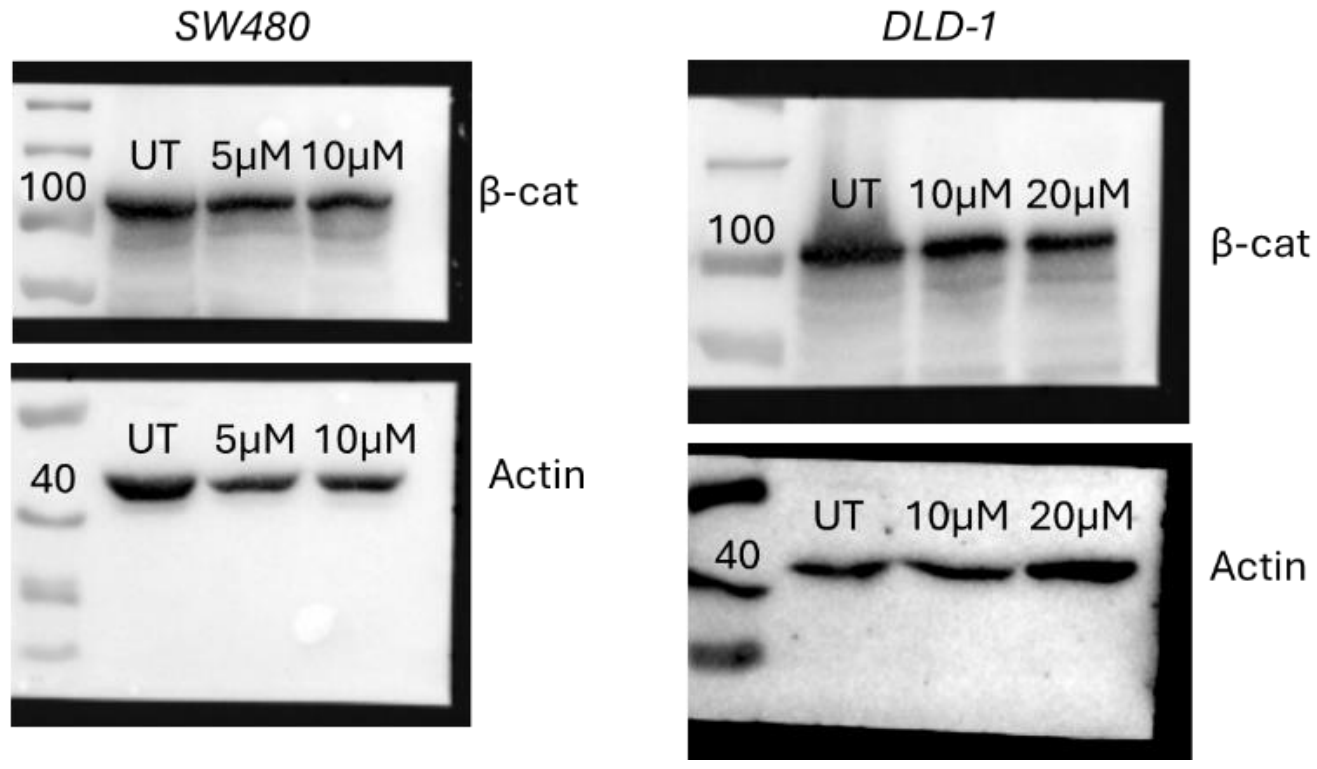

Sup. Figure 2O

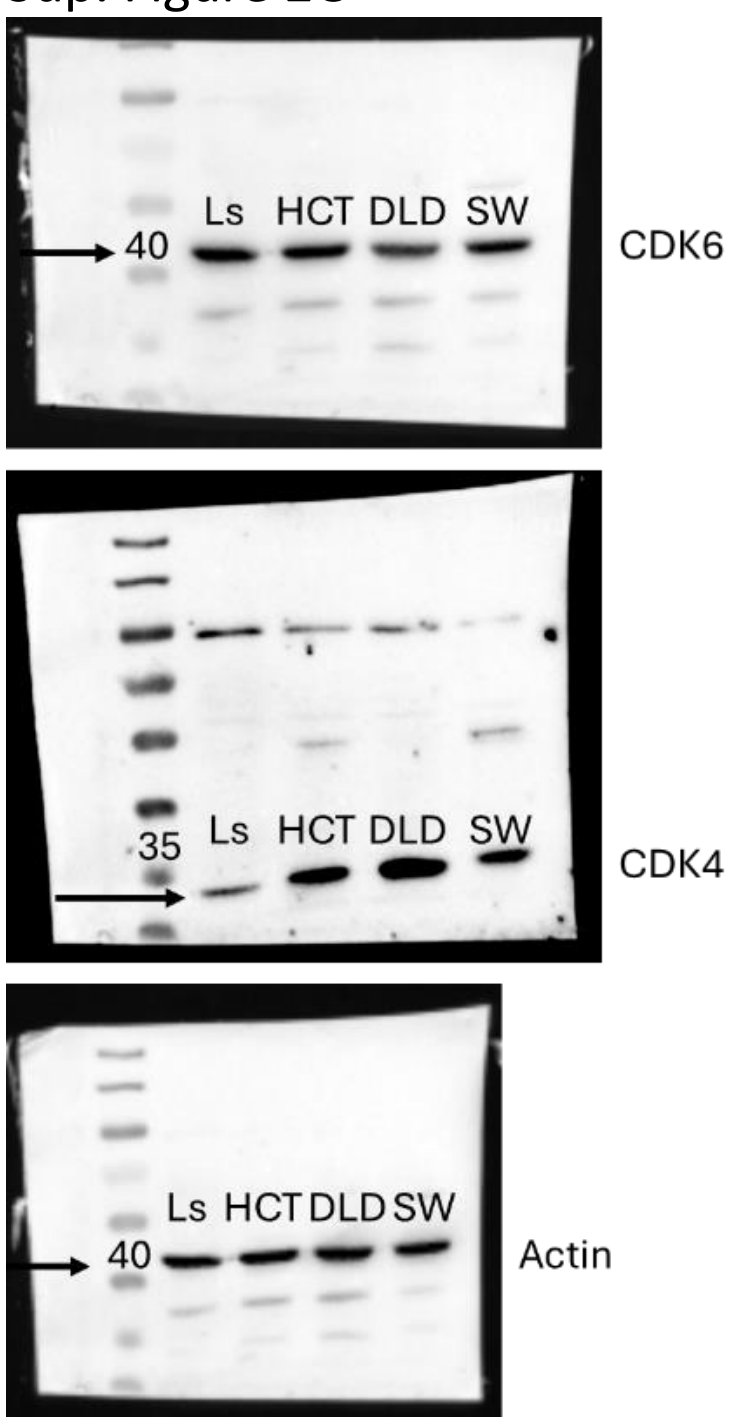

Sup. Figure 3A

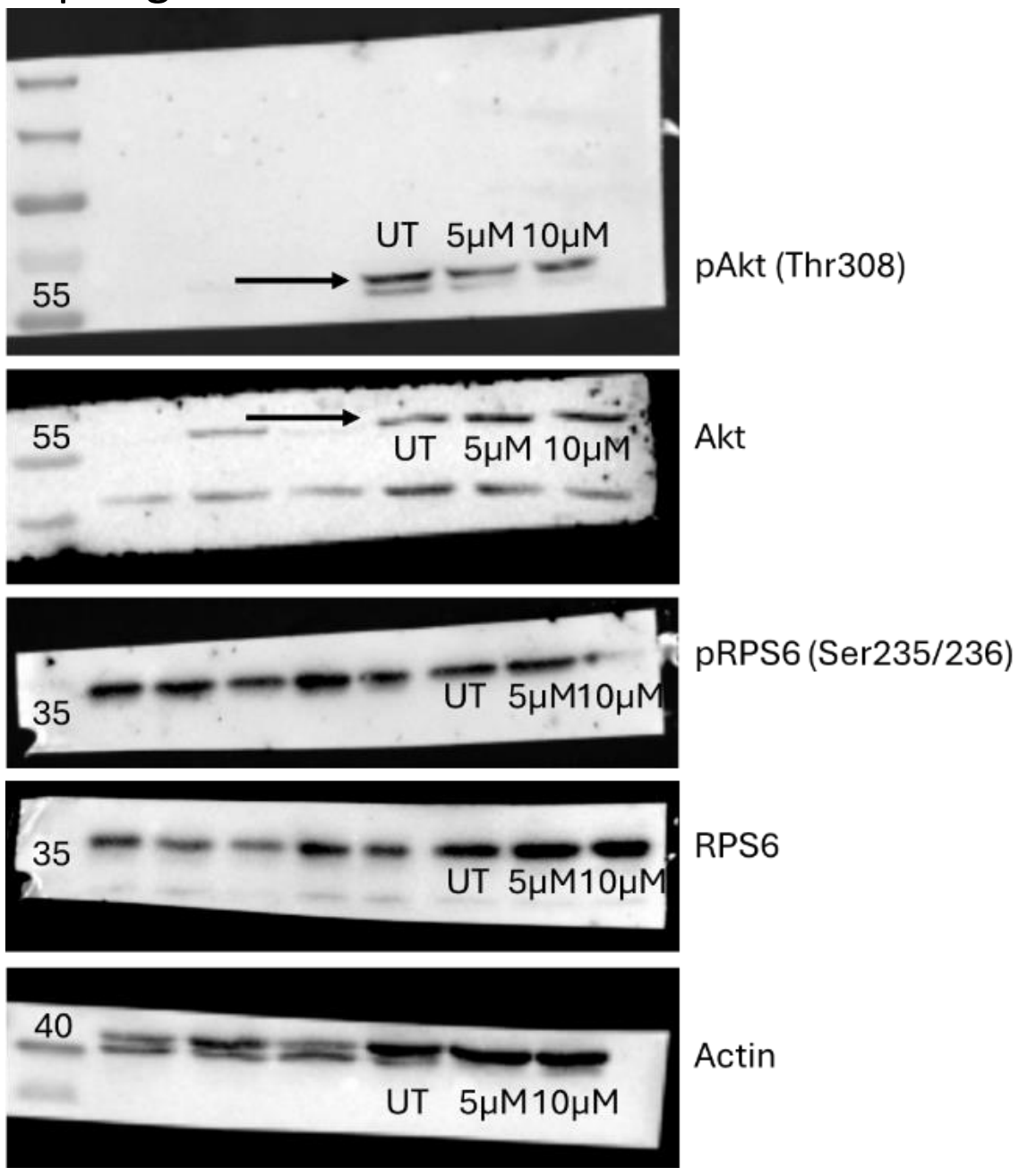

Sup. Figure 3B

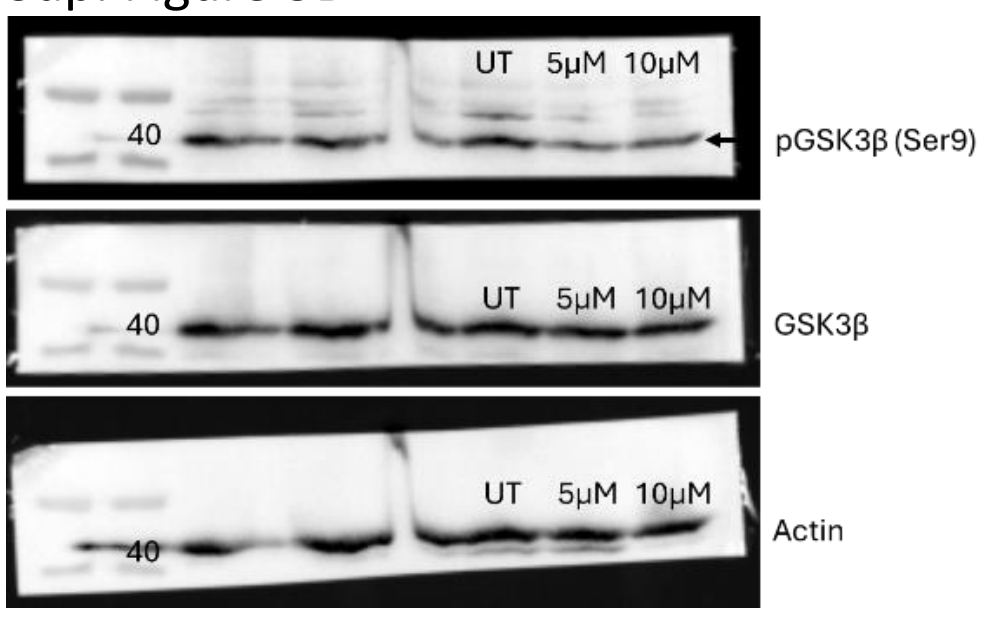

Sup. Figure 3C

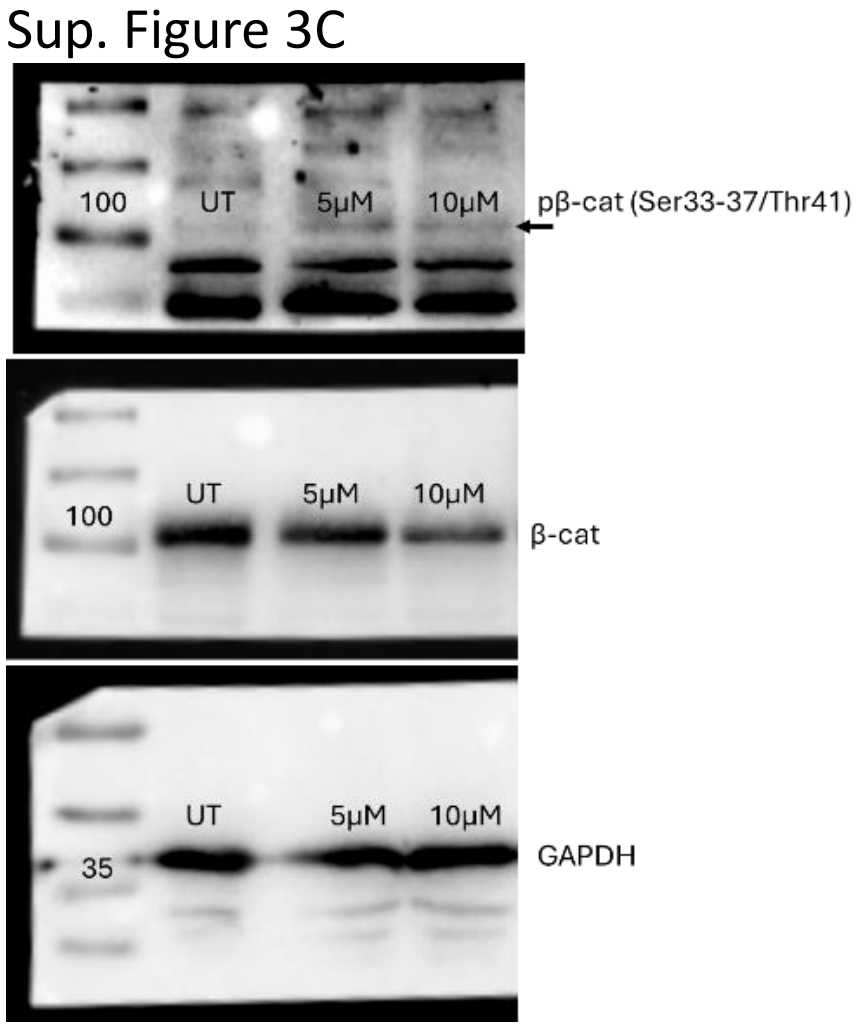

Sup. Figure 3D

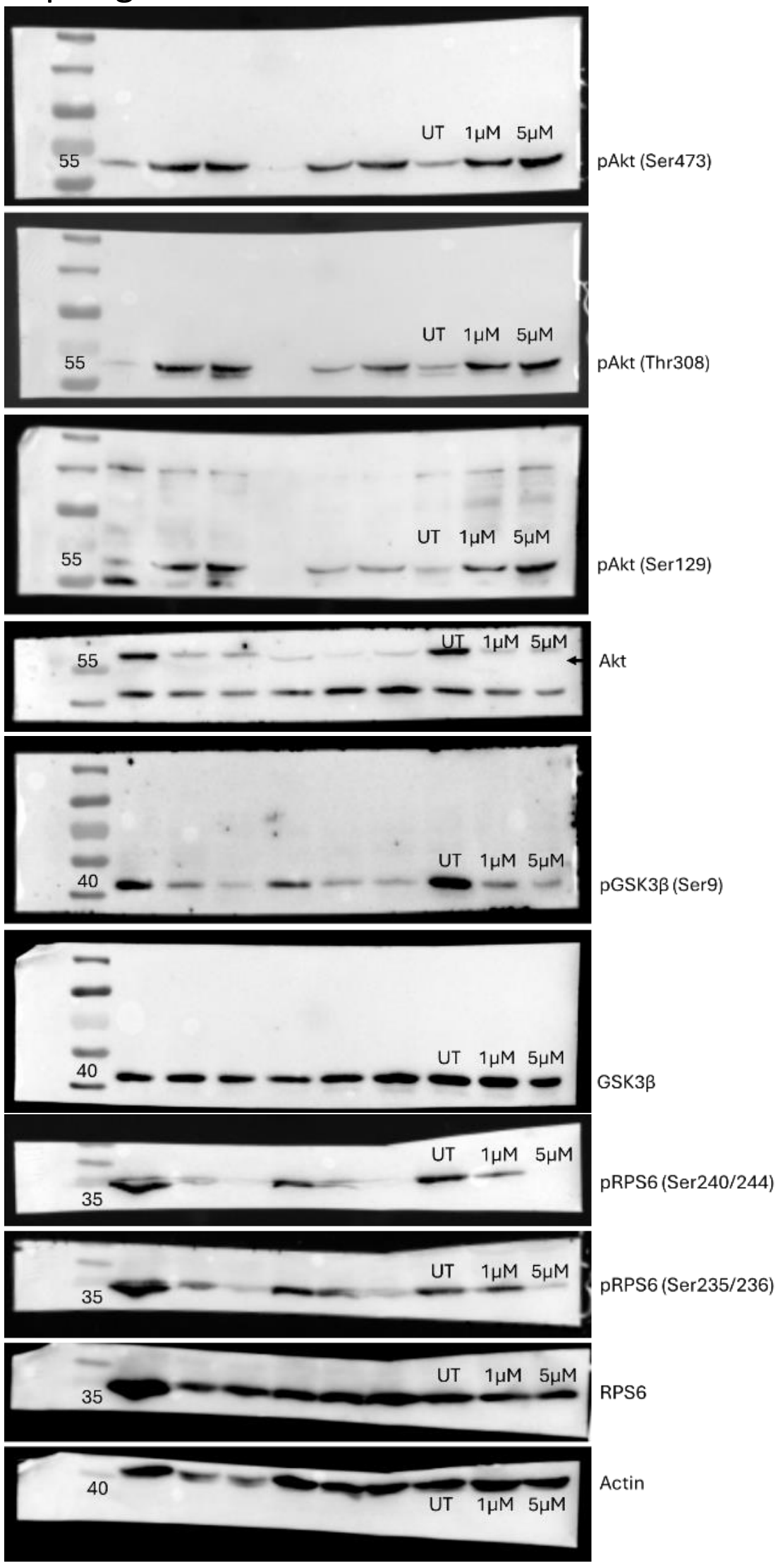

Sup. Figure 3E

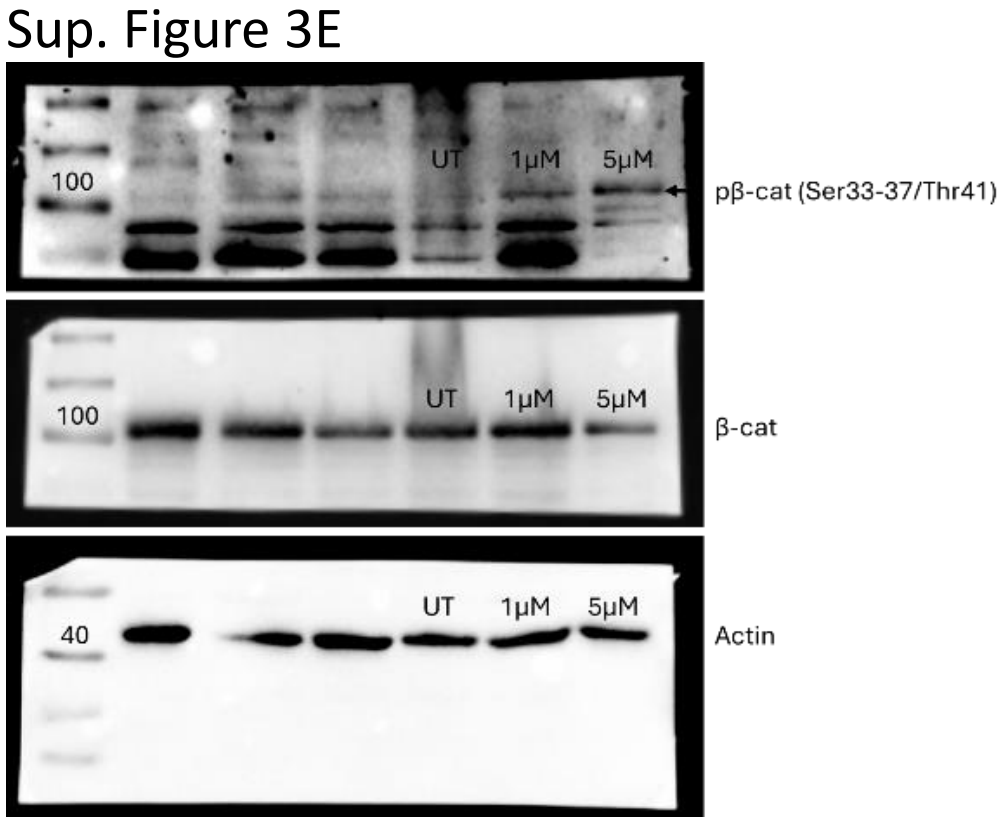

Supplement: Supplementary file 1 [file 12967_2026_8420_MOESM12_ESM.pdf]
