# Supplementary material for: Palbociclib targets β-catenin for degradation and synergizes with KRAS or ERK5 inhibition in colorectal cancer preclinical models
Source: J Transl Med. 2026 Jun 19;24:1132. doi: 10.1186/s12967-026-08420-7 (PMC13540963; doi:10.1186/s12967-026-08420-7)
Supplement: Supplementary file 2 [file 12967_2026_8420_MOESM10_ESM.docx]

**Supplementary Table 3.** Activity of Tucatinib and Palbociclib on CRC and control cells, when used as single agents. Percent viability is reported. The expected Bliss additive effects were calculated from this data.

|  | **Normalized cell growth (%)** | | | |
| --- | --- | --- | --- | --- |
|  | **Ls174T** | | **RWPE1** | |
| **Compounds** | **1µM** | **3µM** | **1µM** | **3µM** |
| Tucatinib | 53.9 | 57.2 | 100 | 100 |
| Palbociclib | 62.6 | 45.1 | 100 | 100 |
