# Supplementary material for: Palbociclib targets β-catenin for degradation and synergizes with KRAS or ERK5 inhibition in colorectal cancer preclinical models
Source: J Transl Med. 2026 Jun 19;24:1132. doi: 10.1186/s12967-026-08420-7 (PMC13540963; doi:10.1186/s12967-026-08420-7)
Supplement: Supplementary file 3 [file 12967_2026_8420_MOESM11_ESM.docx]

**Supplementary Table 4.** Activity of the 6 compounds evaluated for synergistic combination, on CRC and control cells, when used as single agents. Percent viability is reported. The expected Bliss additive effects were calculated from this data.

|  | **Normalized cell growth**  **(% of control)** | | | |
| --- | --- | --- | --- | --- |
|  | **Ls174T** | | **RWPE1** | |
| **Compound** | **1µM** | **10µM** | **1µM** | **10µM** |
| Tucatinib | 58 | 33 | 100 | 100 |
| Palbociclib | 64 | 15 | 90 | 100 |
| LRRK2-IN-1 | 85 | 16 | 100 | 89 |
| ERK5-IN-1 | 88 | 7.5 | 100 | 89 |
| BI-D1870 | 100 | 28 | 100 | 100 |
| Vacquinol-1 | 100 | 1.5 | 85 | 69 |
