# Supplementary material for: Palbociclib targets β-catenin for degradation and synergizes with KRAS or ERK5 inhibition in colorectal cancer preclinical models
Source: J Transl Med. 2026 Jun 19;24:1132. doi: 10.1186/s12967-026-08420-7 (PMC13540963; doi:10.1186/s12967-026-08420-7)
Supplement: Supplementary file 4 [file 12967_2026_8420_MOESM8_ESM.docx]

**Supplementary Table 1.** Relevant mutations in the CRC cell lines used in this study.

| Cell Line | APC | CTNNB1 | KRAS | BRAF | TP53 |
| --- | --- | --- | --- | --- | --- |
| **Ls174T** | wt | S45F | G12D | wt | wt |
| **HCT-116** | wt | S45del | G13D | wt | wt |
| **DLD-1** | G1416fs | wt | G13D | wt | S241F |
| **SW480** | Q1338* | wt | G12V | wt | R273H/P309S |
| **SW837** | R1450*/R213* | wt | G12C | wt | R248W |
