# Supplementary material for: Palbociclib targets β-catenin for degradation and synergizes with KRAS or ERK5 inhibition in colorectal cancer preclinical models
Source: J Transl Med. 2026 Jun 19;24:1132. doi: 10.1186/s12967-026-08420-7 (PMC13540963; doi:10.1186/s12967-026-08420-7)
Supplement: Supplementary file 5 [file 12967_2026_8420_MOESM9_ESM.docx]

**Supplementary Table 2.** Activity of 15 compounds passing screening phase 2, on CRC and control cells, and on Ls174T cells ±doxycycline. IC_50_ values of 72h viability assays are reported in nanomolar units. The activity ratio is shown (phase 2: RWPE1/Ls174T; phase 3: +dox/-dox).

|  | **IC50 (nM)** | | | | | |
| --- | --- | --- | --- | --- | --- | --- |
|  | ***Phase 2*** | | | ***Phase 3*** | | |
| **Compound** | **Ls174T** | **RWPE1** | ***Ratio*** | **+Dox** | **-Dox** | ***Ratio*** |
| Tucatinib  (Irbinitinib, ARRY-380, ONT-380) | 1580 | >10000 | *>6.33* | 16940 | 2389 | *7.09* |
| Miransertib (ARQ 092) HCl | 3556 | >10000 | *>2.81* | 6684 | 1857 | *3.60* |
| Palbociclib (PD-0332991) HCl | 5285 | >10000 | *>1.89* | 5185 | 1614 | *3.21* |
| Uprosertib (GSK2141795) | 1533 | >10000 | *>6.52* | 4043 | 1701 | *2.38* |
| RN486 | 1812 | 8532 | *4.71* | 6868 | 3573 | *1.92* |
| SU9516 | 2943 | 8246 | *2.80* | 8060 | 4358 | *1.85* |
| LRRK2-IN-1 | 3803 | >10000 | *>2.63* | 5978 | 3324 | *1.80* |
| BI-78D3 | 356 | 1277 | *3.59* | 2290 | 1497 | *1.53* |
| Dovitinib (TKI-258) Dilactic Acid | 2035 | 7188 | *3.53* | 6970 | 4902 | *1.42* |
| ERK5-IN-1 (XMD17-109) | 4856 | >10000 | *>2.06* | 3620 | 2802 | *1.29* |
| Momelotinib (CYT387) | 6010 | >10000 | *>1.66* | 9523 | 8652 | *1.10* |
| MNS (MDBN) | 1391 | 4360 | *3.13* | 7341 | 7054 | *1.04* |
| BI-D1870 | 4403 | >10000 | *>2.27* | 8291 | 8460 | *0.98* |
| Vacquinol-1 | 3863 | >10000 | *>2.59* | 6867 | 7027 | *0.98* |
| CP-673451 | 2889 | 9864 | *3.41* | 7159 | 7503 | *0.95* |
